# Extramedullary versus intramedullary fixation of unstable trochanteric femoral fractures (AO type 31-A2): a systematic review and meta-analysis

**DOI:** 10.1007/s00402-023-05138-9

**Published:** 2024-01-04

**Authors:** Miliaan L. Zeelenberg, A. Cornelis Plaisier, Leendert H. T. Nugteren, Sverre A. I. Loggers, Pieter Joosse, Michiel H. J. Verhofstad, Dennis Den Hartog, Esther M. M. Van Lieshout, STABLE-HIP Study Group, Taco Gosens, Johannes H. Hegeman, Suzanne Polinder, Rudolf W. Poolman, Hanna C. Willems, Rutger G. Zuurmond

**Affiliations:** 1 Trauma Research Unit Department of Surgery, Erasmus MC, University Medical Center Rotterdam, P.O. Box 2040, 3000 CA Rotterdam, The Netherlands; 2 Department of Surgery, Noordwest Ziekenhuisgroep, Alkmaar, The Netherlands

**Keywords:** Trochanteric, Hip fracture, Intramedullary, Extramedullary, AO type 31-A2

## Abstract

**Objective:**

The aim of this systematic review was to compare extramedullary fixation and intramedullary fixation for AO type 31-A2 trochanteric fractures in the elderly, with regard to functional outcomes, complications, surgical outcomes, and costs.

**Methods:**

Embase, Medline, Web of Science, Cochrane Central Register of Controlled Trials, and Google Scholar were searched for randomized controlled trials (RCTs) and observational studies. Effect estimates were pooled across studies using random effects models. Results are presented as weighted risk ratio (RR) or weighted mean difference (MD) with corresponding 95% confidence interval (95% CI).

**Results:**

Fourteen RCTs (2039 patients) and 13 observational studies (22,123 patients) were included. Statistically superior results in favor of intramedullary fixation were found for Harris Hip Score (MD 4.09, 95% CI 0.91–7.26, *p* = 0.04), Parker mobility score (MD − 0.67 95% CI − 1.2 to − 0.17, *p* = 0.009), lower extremity measure (MD − 4.07 95% CI − 7.4 to − 0.8, *p* = 0.02), time to full weight bearing (MD 1.14 weeks CI 0.92–1.35, *p* < 0.001), superficial infection (RR 2.06, 95% CI 1.18–3.58, *p* = 0.01), nonunion (RR 3.67, 95% CI 1.03–13.10, *p* = 0.05), fixation failure (RR 2.26, 95% CI 1.16–4.44, *p* = 0.02), leg shortening (MD 2.23 mm, 95% CI 0.81–3.65, *p* = 0.002), time to radiological bone healing (MD 2.19 months, 95% CI 0.56–3.83, *p* = 0.009), surgery duration (MD 11.63 min, 95% CI 2.63–20.62, *p* = 0.01), operative blood loss (MD 134.5 mL, 95% CI 51–218, *p* = 0.002), and tip-apex distance > 25 mm (RR 1.73, 95% CI 1.10–2.74, *p* = 0.02). No comparable cost/costs-effectiveness data were available.

**Conclusion:**

Current literature shows that several functional outcomes, complications, and surgical outcomes were statistically in favor of intramedullary fixation when compared with extramedullary fixation of AO/OTA 31-A2 fractures. However, as several of the differences found appear not to be clinically relevant and for many outcomes data remains sparse or heterogeneous, complete superiority of IM fixation for AO type 31-A2 fractures remains to be confirmed in a detailed cost-effectiveness analysis.

**Supplementary Information:**

The online version contains supplementary material available at 10.1007/s00402-023-05138-9.

## Introduction

Proximal femoral fractures are a major health problem among the elderly worldwide and the incidence rate is rising due to progressive aging. It is expected that the total number of patients with a proximal femoral fracture will rise from 1.66 million in 1990 to 6.26 million worldwide by 2050 [1–3]. These fractures are not exclusively a problem for public health systems, but they also form a burden for society, due to high disability, costs, and morbidity [4–8].

Trochanteric fractures make up 33–52% of the total number of proximal femoral fractures [9, 10]. They are subdivided into stable (two-part) trochanteric with intact lateral wall (31-A1), unstable multi-fragmentary trochanteric with incomplete lateral wall (31-A2), and unstable intertrochanteric (reverse obliquity) fractures (31-A3) by the Arbeitsgemeinschaft für Osteosynthesefragen/Orthopaedic Trauma Association (AO/OTA) classification [11]. Of all proximal femoral fractures, 18–20% are classified as 31-A2 trochanteric fractures [12]. Surgical treatment options for trochanteric fractures are either fixation using an intramedullary nail or extramedullary fixation using plates with or without a sliding hip screw.

Current surgical guidelines such as the United Kingdom’s National Institute for Health and Care Excellence (NICE) guideline and the Dutch Guideline for treatment of proximal femoral fractures advise the use of extramedullary fixation for both AO type 31-A1 and 31-A2 fractures, mainly due to better cost-effectiveness [13, 14]. Extramedullary fixation is more cost effective than intramedullary fixation in the majority of cases, largely because of lower implant costs [15]. The guidelines do, however, see both fixation strategies as viable treatment options for type 31-A2 fractures and underline the absence of conclusive evidence of superiority for either device. Older literature, mainly utilizing the now obsolete first generation of cephalomedullary nails, discouraged intramedullary devices due to a higher failure and reoperation rate [16].

While clear historic evidence of superiority is missing, in recent years the use of intramedullary fixation is rising to up to 90% in the U.S. [17, 18]. While more recent studies report improving outcomes for intramedullary fixation, the optimal treatment strategy remains a topic of debate and the increasing trend of intramedullary fixation may be caused by other factors than clinical data alone [17–21].

Therefore, the objective of this study was to compare recent literature assessing the differences in functional outcomes, complications, surgical outcomes, and costs/cost-effectiveness between extramedullary and intramedullary fixation using currently available implants in elderly patients with AO type 31-A2 fractures.

## Methods

This systematic review and meta-analysis was reported according to the Preferred Reporting Items for Systematic Reviews and Meta-Analysis (PRISMA) guidelines [22]. A protocol was developed prior to conducting the current study. This study did not require approval from the local medical research ethics committee.

### Search and eligibility criteria

Embase, PubMed/Medline, Web of Science, Cochrane Central Register of Controlled Trials, and Google Scholar were searched on 22 March 2021 and updated on 26 September 2022, including terms related to ‘trochanteric fractures,’ ‘intramedullary treatment,’ and ‘extramedullary treatment.’ An overview of the complete search used is included in Online Resource 1. After deduplication, two reviewers (LHTN and ACP) independently screened all articles for eligibility by title and abstract. Thereafter, independent full-text analysis for eligibility was conducted. Disagreements were resolved by consensus.

Studies were included when they presented data (a) published after 1990 of (b) acute (c) AO 31-A2 trochanteric fractures, (d) comparing intramedullary (IM) and extramedullary (EM) fixation (e) in patients aged 50 years and older, (f) using currently available devices. Studies were excluded when they (a) presented no original data, (b) did not mention relevant outcomes, (c) were biomechanical, in vitro or cadaveric studies, (d) pathological fractures, (e) bilateral fractures, (f) peri-implant fractures, (g) were case reports, and (h) did not make distinction between types of fracture or treatment.

### Quality assessment

The included studies were assessed by two reviewers (LHTN and ACP) independently, using the Cochrane Risk of Bias tool, version 2 (RoB2) for the randomized controlled trials (RCTs) and the methodological index for nonrandomized studies (MINORS) for the observational studies [23, 24]. RoB2 provides a risk of bias judgement resulting in low, some concerns, or high risk of bias. The MINORS provides a score with a maximum of 16 points for noncomparative studies and 24 points for comparative studies. A higher score indicates higher quality.

### Data collection

Data from all included studies were independently extracted by two reviewers (LHTN and ACP) according to a predefined data sheet. The baseline characteristics collected for each study contained the following: first author, year of publication, setting (country), inclusion period, study design, type of fixation device used, follow-up period, and mean age, gender distribution, and total number of patients with AO-OTA 31-A2 fracture in the study population. See Online Resource 2 for specific devices used per included study.

Data were collected on the following outcome measures: Functional outcomes: Harris hip score (HHS), Parker mobility score, lower extremity measure (a modification of the Toronto extremity salvage score (TESS) [25]), recovery to pre-operative walking ability, and time to full weight bearing; Complications: reoperation, superficial wound infection, deep wound infection, nonunion, cut-out/protrusion (varus collapse of the neck-shaft angle leading to extrusion of the screw), peri-prosthetic fracture, conversion to prosthesis, implant/fixation failure (mechanical loosening or fracturing of the implant), heterotopic ossification, leg shortening, screw migration, femur shaft fracture, and mortality; Surgical outcomes/operation characteristics: mean time to bone healing, radiologic quality of reduction, surgery duration, hospital stay, blood loss, blood transfusion (units per patient), blood transfusion, fluoroscopy time, tip-apex distance (TAD), TAD > 25 mm, femoral neck shortening, and neck-shaft angle (NSA); and Costs/cost-effectiveness. All outcome measures were included and analyzed as defined by the original article.

Authors of studies with missing data (standard deviations) were contacted by email once [26–30]. When no data were received, standard deviations were imputed by weighted mean SD of the other included studies, if at least two other studies were available.

### Statistical analysis

RevMan version 5.4 was used to analyze data. Binary outcomes were pooled using the Cochran–Mantel–Haenszel statistic and presented as risk ratio (RR) and continuous outcomes were pooled using the inverse variance weighting method and presented as mean difference (MD), both with corresponding 95% confidence intervals (95% CI). All analyses were done separately for each of the different study designs (RCTs and observational studies) and for overall effect and were presented in Forest plots. Random effects models were used in all comparisons because of a high likelihood of heterogeneity between studies due to inclusion of both RCT and observational studies and comparison of multiple types of devices in different countries and clinical settings. Assessment of heterogeneity between studies was done by using the Cochrane Q-test and was quantified using the *I*^2^ statistic. A *p*-value < 0.05 was considered statistically significant. According to the Cochrane Handbook for Systematic Reviews of Interventions, the level of heterogeneity was found to be unimportant when the *I*^2^ value is between 0 and 40%, moderate between 30 and 60%, substantial between 50 and 90%, and considerable between 75 and 100% [31]. Funnel plots were visually inspected to assess publication bias (Online Resource 3).

## Results

### Search

**Fig. 1:**
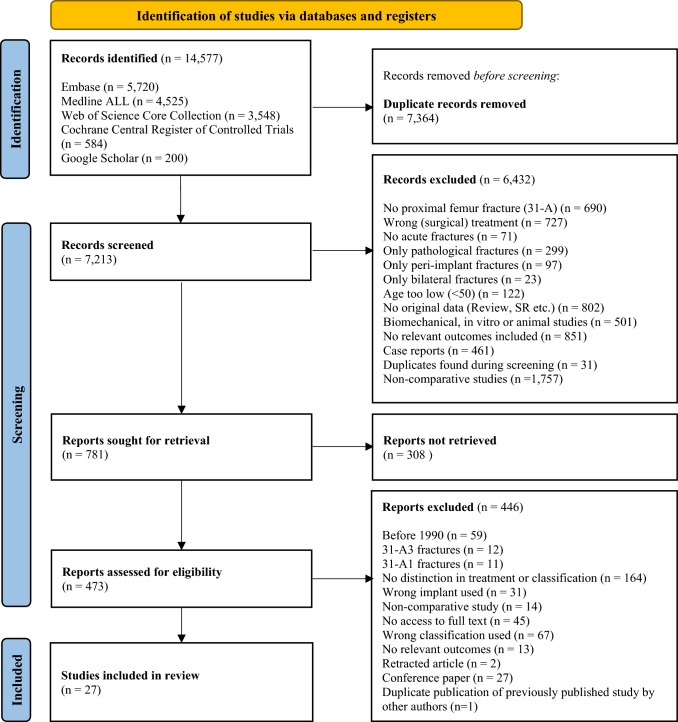
Flowchart of search results, article inclusion, and exclusion

The primary search resulted in a total of 14,577 records. After deduplication 7213 studies remained for screening (Fig. 1). Out of 473 full-text articles assessed for eligibility, 27 were included in this systematic review and meta-analysis. Fourteen were RCTs [26, 28, 30, 32–42] and 13 were observational studies [19, 20, 27, 29, 43–51].

### Study characteristics

The included studies were published from 2006 to 2022 and provided data of 24,232 patients with an AO-OTA 31-A2 trochanteric fracture, of whom 2039 patients were included in RCTs and 22,123 in observational studies (Table 1). Of all included patients, 11,932 were treated with an extramedullary (EM) device and 12,300 with an intramedullary (IM) device. Table S2 in Online resource 4 (OR4) provides an overview of the outcome measures reported in the individual studies. The mean follow-up time was 12 months.

**Table 1:** Overview of included studies comparing intramedullary versus extramedullary fixation in AO type 31-A2 fractures

Study (publication date)	Country	Study design	Inclusion period	Total population AO type 31-A2 fracture	Extramedullary fixation				Intramedullary fixation				Follow-up (months)
					Number of patients	Device	Age (years)	Males (%)	Number of patients	Device	Age (years)	Males (%)	
*Randomized controlled trials*													
Aktselis et al. [32]	Greece	RCT	2008–2011	71	35	AMBI DHS	83.1	20.0	36	G3 Nail	82.9	22.2	12
Andalib et al. [33]	Iran	RCT	2016–2018	93	55	DHS, DCS	61.45	47.2	38	CMN	64.40	44.7	12
Barton et al. [26]	United Kingdom	RCT	N.D	210	110	SHS	83.3	22.7	100	Long GN	83.2	19.0	12
Garg et al. [42]	India	RCT	2013–2018	105*	35	DHS	69.8	31.4	35	PFN	70.38	28.6	12
Ovesen et al. [38]	Denmark	RCT	2001–2003	96	52	DHS	78.5^a^	28.8^a^	44	TGN	79.9^a^	27.4^a^	12
Pajarinen et al. [39]	Finland	RCT	1999–2001	50	24	DHS	80.3^a^	25.9^a^	26	PFN	80.9^a^	24.1^a^	4
Parker et al. [40]	United Kingdom	RCT	2002–2009	717	364	SHS	82.1^a^	23.2^a^	353	IN	82.2^a^	22.4^a^	12
Reindl et al. [28]	Canada	RCT	N.D	204	92	DHS	80	33.7	112	Intertan, GN	82	51.0	12
Saleem et al. [34]	Pakistan	RCT	2017–2019	108	54	DHS	60.2	68.5	54	PFN	58.54	57.4	5.5
Singh et al. [35]	India	RCT	2009–2011	26	12	PF-LCP	60.5^a^	39.1^a^	14	PFN	58.3^a^	31.8^a^	24
Tao et al. [41]	China	RCT	2010–2011	42	21	Reverse LISS	80.7	23.8	21	PFNA	82.5	52.4	12
Verettas et al. [30]	Greece	RCT	N.D	118	59	DHS	79.22	25.4	59	IMN	81.03	33.9	0.3
Xu et al. [36]	China	RCT	2006–2008	106	55	DHS	77.9	29.1	51	PFNA	78.5	29.4	12
Zehir et al. [37]	Turkey	RCT	2010–2013	198	102	DHS	78.86	38.2	96	PFNA	77.22	38.5	15.95^c^
*Observational studies*													
Andruszkow et al. [43]	Germany	Retrospective cohort	2007–2010	120	86	DHS	80.8^a,b^	29.8^a,b^	34	GN	80.8^a,b^	29.8^a,b^	N.D
Butt et al. [44]	United Kingdom	Retrospective cohort	2015–2016	79	50	DHS	74.8^a^	29.8^a,b^	29	PFNA	80.6^a^	29.8^a,b^	N.D
Crespo et al. [45]	Spain	Retrospective cohort	2004–2009	235	115	PCCP	82.5^a^	26.7^a^	120	GN	83.1^a^	18.4^a^	12
Duymus et al. [46]	Turkey	Retrospective cohort	2012–2014	91	30 + 29	DHS + PF-LCP	70.68	44.1	32	PFN	71.66	40.6	EM: 26.22; IM: 25.77
Gronhaug et al. [20]	Norway	Prospective cohort	2013–2019	7168	4193	SHS	83.5	26.5	2975	IMN	83.3	26.4%	36
Knobe et al. [27]	Germany	Retrospective cohort	2002–2007	135	36 + 46	DHS + PCCP	78.3	31.7	53	PFN	77.2	39.6	EM: 16.2; IM: 19.2
Knobe et al. [47]	Germany	Prospective cohort	2005–2008	108	54	PCCP	81	24.1	54	PFNA	78	24.1	24
Müller et al. [48]	Germany	Retrospective cohort	2006–2015	375	75 + 100	DHS + AR (+ TSP)	83.5	21.1	200	PFNA	82.6	23.0	24
Page et al. [49]	United Kingdom	Retrospective cohort	2011–2015	370	267	DHS	85.90	27.7	103	IMN	86.03	18.4	N.D
Pyrhonen et al. [19]	Sweden	Retrospective cohort	2012–2018	10,213	3,187	SHS	85^d^	28.8	7,026	IMN	85^d^	27.7	60
Sevinç et al. [29]	Turkey	Prospective cohort	N.D	58	18	DHS	77.1^a^	59.1^a^	40	PFNA	78.9^a^	48.2^a^	12
Suh et al. [50]	Korea	Retrospective cohort	2010–2012	100	50	CHS	77.3	42	50	PFNA	73.8	48	12
Tucker et al. [51]	United Kingdom	Prospective cohort	2000–2015	3071	2377 + 149	SHS (+ TSP)	80.2^a,b^	23.5^a,b^	545	CMN	80.2^a,b^	23.5^a,b^	12

*RCT*, Randomized controlled trial; *(AMBI) DHS*, Dynamic Hip Screw; *G3*, Gamma3 Nail; *DCS*, Dynamic condylar screw; *CMN*, Cephalomedullary nail; *SHS*, Sliding hip screw; *GN*, Gamma nail; *TGN*, Trochanteric gamma nail; *PFN*, Proximal femoral nail; *PFNA*, Proximal femoral nail antirotation; *IN*, Intramedullary nail; *PF-LCP*, Proximal femoral locking compression plate; *Reverse LISS*, Reverse less invasive stabilization system; *IMN*, Intramedullary nail; *PCCP*, Percutaneous compression plate; *ARS*, Antirotation screw; *TFN*, Trochanteric fixation nail; *TSP*, Trochanteric stabilization plate; *CHS*, Compression hip screw; *N.D.*, Not described; *N.S.*, Not specified

*Of the total population, 35 patients were treated by (hemi)arthroplasty

^a^Value for total study population including AO type 31-A1 and/or A3 fractures

^b^Value for individual groups not specified in study

^c^Median follow-up duration

^d^Median age

### Quality assessment

The RoB 2 overall bias assessment indicated high risk of bias in three RCTs [30, 33, 39], medium risk in six RCTs [28, 32, 36, 40, 42, 52], and low risk in five RCTs [26, 34, 37, 38, 41] (OR4, Table S3). The MINORS score for observational studies ranged from a minimum of 13 [44] (moderate quality) to a maximum of 22 [47] (high quality) with a mean of 17 (SD 2.5) (OR4, Table S4).

### Functional outcomes

#### Harris hip score (HHS)

**Fig. 2:**
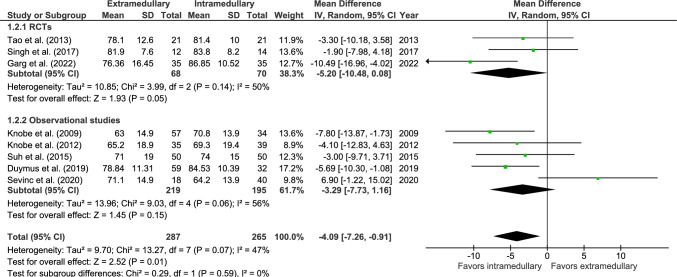
Forest plot of Harris Hip Score after extramedullary versus intramedullary fixation of AO 31-A2 fractures. Standard deviations for Knobe et al. and Sevinc et al. [27, 29] were imputed. IV, inverse variance; RCT, randomized controlled trial; SD, standard deviation

The Harris hip score at one-year follow-up was reported in eight studies, three RCTs [35, 41, 42] and five observational studies [27, 29, 46, 47, 50], in which 265 patients were treated with EM fixation and 287 patients with IM fixation (Fig. 2). The mean HHS was 72 for EM fixation and 75 for IM fixation. The HHS significantly favored IM fixation (MD − 4.09, 95% CI − 7.26 to − 0.91, *p* = 0.01, *I*^2^ = 47%).

#### Parker mobility score (PMS)

**Fig. 3:**
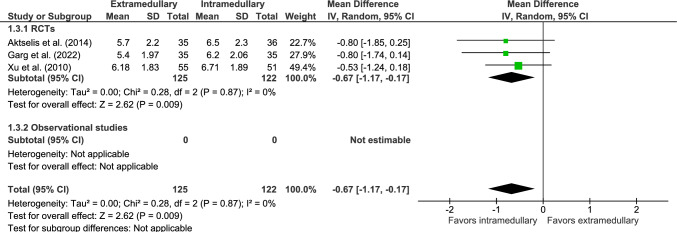
Forest plot of Parker mobility score after extramedullary versus intramedullary fixation of AO 31-A2 fractures. Standard deviations for Garg et al. [42] were imputed

Parker mobility score at one-year follow-up was reported in three RCTs (Fig. 3) [32, 36, 42], in which 125 patients were treated with EM fixation and 122 with IM fixation. The mean PMS was 5.8 for EM devices and 6.5 for IM devices. The PMS significantly favored IM fixation (MD − 0.67, 95% CI − 1.2 to − 0.17, *p* = 0.009, *I*^2^ = 0%).

#### Lower extremity measure (LEM)

Lower extremity measure at one-year follow-up was reported in two RCTs (OR4, Figure S2) [28, 33], in which 135 patients were treated with EM devices and 125 were treated with IM devices. The mean LEM was 64.5 for EM devices and 67.0 for IM devices. The LEM significantly favored IM fixation (MD − 4.07, 95% CI − 7.39 to − 0.75, *p* = 0.02, *I*^2^ = 0%).

#### Recovery to pre-operative walking ability

Recovery to pre-operative walking ability was reported in two RCTs (OR4, Figure S3) [36, 37]. This was measured at 6 months by Zehir et al. and up to 1 year by Xu et al. Recovery to pre-operative walking ability was achieved in 87 out of 145 (60.0%) patients treated with an EM device and in 100 out of 136 (73.5%) patients treated with an IM device. There was no significant difference between fixation groups (RR 0.80, 95% CI 0.61–1.05, *p* = 0.11, *I*^2^ = 0%).

#### Time to full weight bearing (weeks)

Time to full weight bearing was reported in one RCT and one observational study, with 94 patients treated by extramedullary fixation and 67 by intramedullary fixation (OR4, Figure S4) [42, 46]. Mean time to full weight bearing was 2.5 weeks for EM fixation and 1.4 weeks for IM fixation. It significantly favored IM fixation (MD 1.14, 95% CI 0.92–1.35, *p* < 0.001, *I*^2^ = 51%).

#### Pain scores

Different measurement scales were used for the pain scores, namely, the VAS [27, 30, 50] and HHS pain score [27]. None of the studies reported a significant difference. Meta-analysis of the VAS score was not possible, because two studies out of three did not report SDs, and thus, no imputed SD could be calculated [30, 50].

#### Other functional scores

Multiple other measurement scales are used for the functional outcomes and quality of life, in addition to those previously mentioned: Merle d’Aubigné and Postel score [27, 47], EQ-5D [32], Barthel index [32], functional independence measure (FIM) [28], Coval score [50], and HHS [27]. None of the included studies reported a significant difference between fixation groups. Due to the high diversity in included functional and quality of life scores and/or missing SDs, no meta-analysis was performed.

An overview of functional outcomes is shown in Table 2.

**Table 2:** Overview of functional outcomes

Outcome	Study type	References	OM	Total population EMF	Total population IMF	Mean/cases		Pooled effect (95% CI), *p*-value	*I*^2^ (%)
						EMF	IMF		
Harris hip score	3 RCTs 5 OS	[27, 29, 35, 41, 46, 47, 50]	MD	287	265	72^a^	75^a^	− 4.09 (− 7.26 to − 0.91) ***p* = 0.04**	47
Parker mobility score	3 RCTs	[32, 36]	MD	125	122	5.8^a^	6.5^a^	− 0.67 (− 1.2 to − 0.17) ***p* = 0.009**	0
Lower extremity measure	2 RCTs	[28, 33]	MD	135	125	64.5^a^	67.0^a^	− 4.07(− 7.39 to − 0.75) ***p* = 0.02**	0
Recovery to pre-operative walking ability	2 RCTs	[36, 37]	RR	145	136	87^b^ (60%)	100^b^ (74%)	0.88 (0.61 to 1.05) *p* = 0.11	44
Time to full weight bearing (weeks)	1 RCT 1 OS	[42, 46]	MD	94	67	2.5^a^	1.4	1.14 (0.92 to 1.35) ***p* < 0.001**	51

Statistically significant differences (*p* < 0.05) are denoted as bold

*OM*, Outcome measurement; *EMF*, Extramedullary fixation; *IMF*, Intramedullary fixation; *RCT*, Randomized controlled trial; *OS*, Observational studies; *RR*, Relative risk; *MD*, Mean difference; *N.A.*, Not available

^a^Subgroup mean

^b^Cases reported in subgroup

### Complications

#### Reoperations

**Fig. 4:**
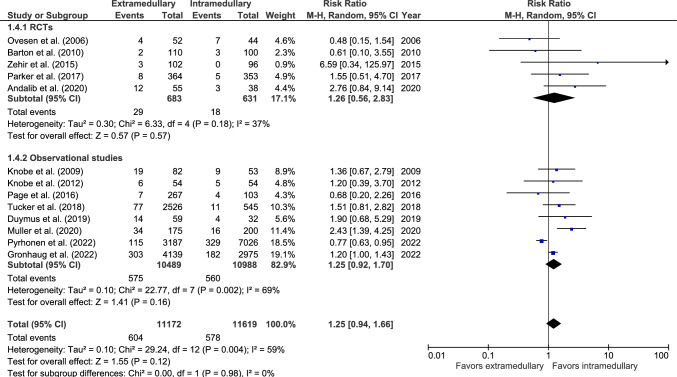
Forest plots of reoperations after extramedullary versus intramedullary fixation of AO 31-A2 fractures. M-H, Mantel–Haenszel; RCT, randomized controlled trial; SD, standard deviation

Reoperation rate was reported in 13 studies: five RCTs [26, 33, 37, 38, 40] and eight observational studies (Fig. 4) [19, 20, 27, 46–49, 51]. Reoperation occurred in 604 out of 11,172 (5.4%) patients treated with an EM device and 578 out of 11,619 (5.0%) treated with an IM device. There was no significant difference between fixation groups (RR 1.25, 95% CI 0.94–1.66, *p* = 0.12, *I*^2^ = 59%).

#### Deep infections

**Fig. 5:**
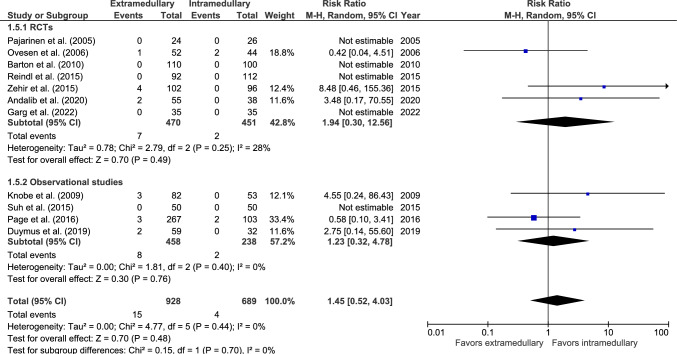
Forest plots of deep infections after extramedullary versus intramedullary fixation of AO 31-A2 fractures

Deep infection rate was reported in 11 studies: seven RCTs [26, 28, 33, 37–39, 42] and four observational studies (Fig. 5) [27, 46, 49, 50]. Deep infection occurred in 15 out of 928 (1.6%) patients treated with an EM device and 4 out of 689 (0.6%) patients treated with an IM device. Five studies reported zero cases [26, 28, 39, 42, 50]. There was no significant difference between fixation groups (RR 1.45, 95% CI 0.52–4.03, *p* = 0.48, *I*^2^ = 0%).

#### Superficial infections

**Fig. 6:**
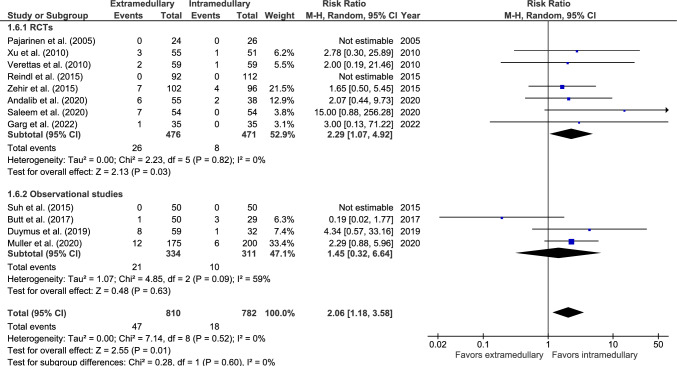
Forest plots of superficial infections after extramedullary versus intramedullary fixation of AO 31-A2 fractures

Superficial infection rate was reported in 12 studies: eight RCTs [28, 30, 33, 34, 36, 37, 39, 42] and four observational studies (Fig. 6) [44, 46, 48, 50]. Superficial infection occurred in 47 out of 810 (5.8%) patients treated with an EM device and 18 out of 782 (2.3%) patients treated with an IM device. Three studies reported zero cases [28, 39, 50]. The risk of superficial infections was significantly lower in the IM group (RR 2.06, 95% CI 1.18–3.58, *p* = 0.01, *I*^2^ = 0%).

#### Nonunion

**Fig. 7:**
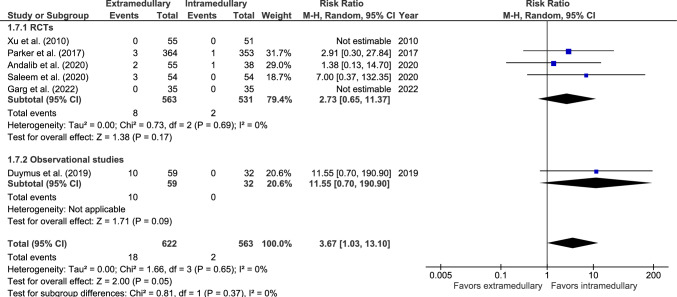
Forest plots of nonunion after extramedullary versus intramedullary fixation of AO 31-A2 fractures

Nonunion rate was reported in six studies: five RCTs [33, 34, 36, 40, 42] and one observational study (Fig. 7) [46]. Nonunion occurred in 18 out of 622 (2.9%) patients treated with an EM device and 2 out of 563 (0.4%) patients treated with an IM device. The risk for nonunion was significantly lower in the IM group (RR 3.67, 95% CI 1.03–13.10, *p* = 0.05, *I*^2^ = 0%).

#### Cut-out/protrusion

**Fig. 8:**
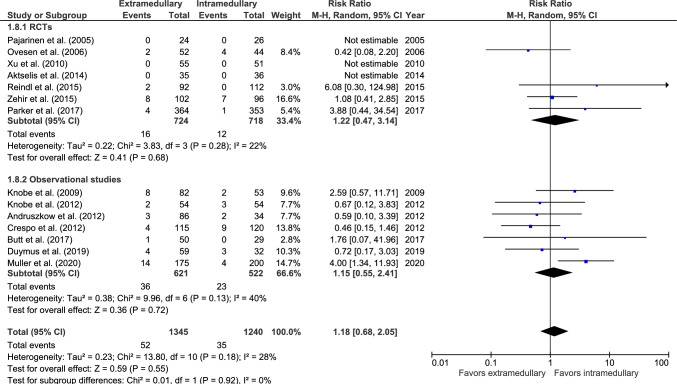
Forest plots of cut-out after extramedullary versus intramedullary fixation of AO 31-A2 fractures

Cut-out rate was reported in fourteen studies: seven RCTs [28, 32, 36–40] and seven observational studies (Fig. 8) [27, 43–48]. Cut-out occurred in 52 out of 1345 (3.9%) patients treated with an EM device and 35 out of 1240 (2.8%) patients treated with an IM device. Three studies [32, 36, 39] reported zero cases. There was no significant difference between fixation groups (RR 1.18, 95% CI 0.68–2.05, *p* = 0.55, *I*^2^ = 28%).

#### Peri-implant fractures

**Fig. 9:**
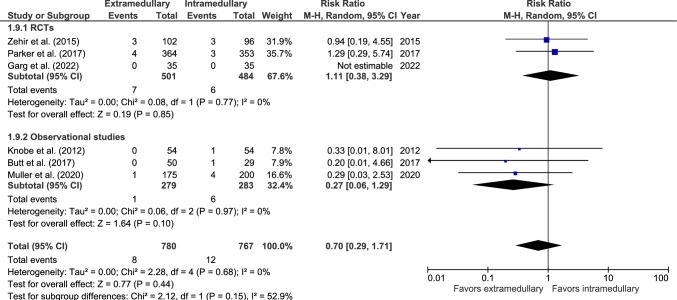
Forest plots of peri-implant fractures after extramedullary versus intramedullary fixation of AO 31-A2 fractures

Peri-implant fracture rate was reported in six studies: three RCTs [37, 40, 42] and three observational studies (Fig. 9) [44, 47, 48]. Peri-implant fracture occurs in 8 out of 780 (1.0%) patients treated with an EM device and 12 out of 767 (1.6%) patients treated with an IM device. Garg et al*.* reported zero cases [42]. There was no significant difference between fixation groups (RR 0.70, 95% CI 0.29–1.71, *p* = 0.44, *I*^2^ = 0%).

#### Conversion to prosthesis

**Fig. 10:**
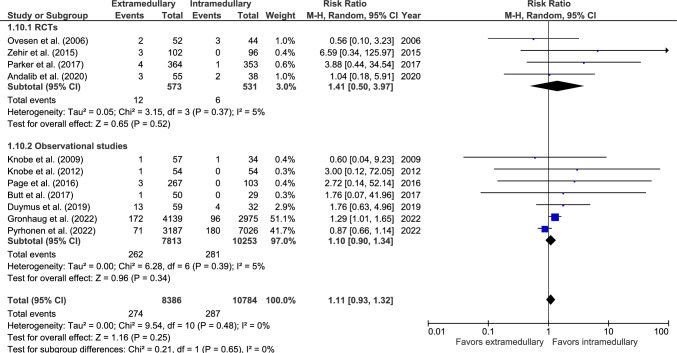
Forest plots of conversion to prosthesis after extramedullary versus intramedullary fixation of AO 31-A2 fractures

Conversion rate was reported in 11 studies: four RCTs [33, 37, 38, 40] and seven observational studies (Fig. 10) [19, 20, 27, 44, 46, 47, 49]. Conversion to prosthesis was reported in 274 out of 8386 (3.3%) patients treated with an EM device and 287 out of 10,784 (1.7%) patients treated with an IM device. There was no significant difference between fixation groups (RR 1.11, 95% CI 0.93–1.32, *p* = 0.25, *I*^2^ = 0%).

#### Fixation failure

**Fig. 11:**
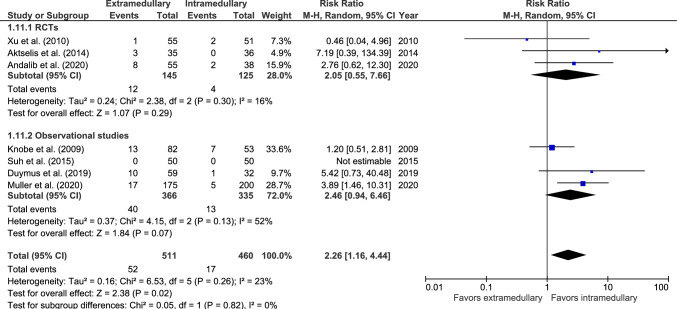
Forest plot of implant/fixation failure after extramedullary versus intramedullary fixation of AO 31-A2 fractures

Fixation/implant failure rate was reported in seven studies: three RCTs [32, 33, 36] and four observational studies (Fig. 11) [27, 46, 48, 50]. Fixation failure occurred in 52 out of 511 (10.2%) patients treated with an EM device and 17 out of 460 (3.7%) patients treated with an IM device. Suh et al. [50] reported zero cases. The risk for fixation failure was significantly lower in the IM group (RR 2.26, 95% CI 1.16–4.44, *p* = 0.02, *I*^2^ = 23%).

#### Heterotopic ossification

Heterotopic ossification was reported in three studies: two RCTs [28, 42] and one observational study [50]. In Reindl et al*.*[28] heterotopic ossification occurred in 23 out of 130 (17.7%) patients treated with an EM device and 49 out of 137 (35.8%) patients treated with an IM device (RR 0.51, 95% CI 0.35–0.76, *p* = 0.008). In respectively, 12 and 35 cases this was Brooker stage 1 heterotopic ossification. No pooled risk ratio for heterotopic ossification could be calculated, because both Garg et al*.* and Suh et al. [42, 50] reported zero cases.

#### Leg shortening (mm)

Leg shortening was reported in four studies: two RCTs [34, 36] and two observational studies (OR4, Figure S5) [47, 50], in which 194 patients were treated with an EM device and 194 with an IM device. The mean leg shortening length was 4.3 mm for EM devices and 2.3 mm for IM devices. Leg shortening was significantly lower for IM fixation (MD 2.23 mm, 95% CI 0.81–3.65, *p* = 0.002, *I*^2^ = 65%).

#### Screw migration

Migration of cephalomedullary screw was reported in three studies: two RCTs [37, 46] and one observational study (OR4, Figure S6) [47]. Screw migration occurred in 10 out of 215 (4.7%) patients treated with an EM device and in 10 out of 182 (5.5%) patients treated with an IM device. There was no significant difference between fixation groups (RR 0.76, 95% CI 0.11–5.08, *p* = 0.77, *I*^2^ = 55%).

#### Femoral shaft fractures

Femoral shaft fractures were reported in two RCTs (OR4, Figure S7) [36, 37]. Femoral shaft fractures occurred in 1 out of 157 (0.6%) patients treated with an EM device and in 4 out of 147 (2.7%) patients treated with an IM device. There was no significant difference between fixation groups (RR 0.38, 95% CI 0.05–2.81, *p* = 0.34, *I*^2^ = 0%).

#### Mortality

**Fig. 12:**
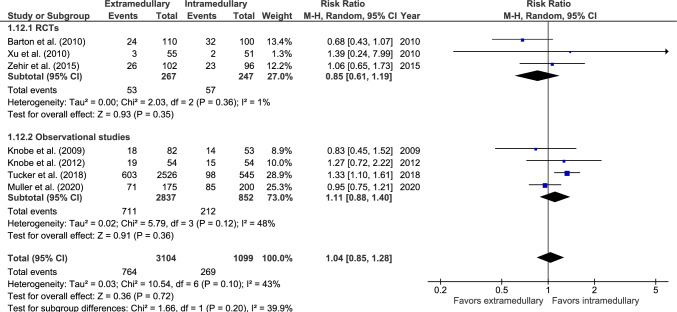
Forest plot of mortality at minimum one-year follow-up after extramedullary versus intramedullary fixation of AO 31-A2 fractures

Mortality rate, after at least one-year follow-up, was reported in seven studies: three RCTs [26, 36, 37] and four observational studies (Fig. 12) [27, 47, 48, 51]. In 764 out of 3104 (24.6%) patients treated with an EM device and 269 out of 1099 (24.5%) patients treated with an IM nail mortality occurred during follow-up. There was no significant difference between fixation groups (RR 1.04, 95% CI 0.85–1.28, *p* = 0.72, *I*^2^ = 43%).

An overview of all complications is given in Table 3.

**Table 3:** Overview of complications

Outcome	Study type	References	OM	Total population EMF	Total population IMF	Mean/cases		Pooled effect (95% CI), *p*-value	*I*^2^ (%)
						EMF	IMF		
Reoperation	5 RCTs 8 OS	[19, 20, 26, 27, 33, 37, 38, 40, 46–49, 51]	RR	11,172	11,619	604^b^ (4.8%)	578^b^ (4.1%)	1.25 (0.94 to 1.66) *p* = 0.12	59
Deep infection	7 RCTs 4 OS	[26–28, 33, 37–39, 42, 46, 49, 50]	RR	928	689	15^b^ (1.6%)	4^b^ (0.6%)	1.45 (0.52 to 4.03) *p* = 0.48	0
Superficial infection	8 RCTs 4 OS	[28, 30, 33, 34, 36, 37, 39, 42, 44, 46, 48, 50]	RR	810	782	47^b^ (5.8%)	18^b^ (2.3%)	2.06 (1.18 to 3.58) ***p* = 0.01**	0
Nonunion	5 RCTs, 1 OS	[33, 34, 36, 40, 42, 46]	RR	622	563	18^b^ (2.9%)	2^b^ (0.4%)	3.67 (1.03 to 13.10) ***p* = 0.05**	0
Cut-out	7 RCTs, 7 OS	[27, 28, 32, 36–40, 43–48]	RR	1345	1240	52^b^ (3.9%)	35^b^ (2.8%)	1.18 (0.68 to 2.05) *p* = 0.55	28
Peri-implant fracture	3 RCTs 3 OS	[37, 40, 44, 47, 48]	RR	780	767	8^b^ (1.0%)	12^b^ (1.6%)	0.70 (0.29 to 1.71) *p* = 0.44	0
Conversion to prosthesis	4 RCTs 7 OS	[19, 20, 27, 33, 37, 38, 40, 44, 46, 47, 49]	RR	8386	10,784	274^b^ (3.3%)	287^b^ (2.7%)	1.11 (0.93 to 1.32) *p* = 0.25	0
Fixation failure	3 RCTs 4 OS	[27, 32, 33, 36, 46, 48, 50]	RR	511	460	52^b^ (10.2%)	17^b^ (3.7%)	2.26 (1.16 to 4.44) ***p* = 0.02**	23
Heterotopic ossification	2 RCT 1 OS	[28, 42, 50]	N.A	167	172	23^b^ (13.8%)	49^b^ (28.5%)	N.A	N.A
Leg shortening (mm)	2 RCTs 2 OS	[34, 36, 47, 50]	MD	194	194	4.3^a^	2.3^a^	2.23 (0.81 to 3.65) ***p* = 0.002**	65
Screw migration	2 RCTs 1 OS	[37, 46, 47]	RR	215	182	10^b^ (4.7%)	10^b^ (5.5%)	0.76 (0.11 to 5.08) *p* = 0.77	55
Femoral shaft fracture	2 RCTs	[36, 37]	RR	157	147	1^b^ (0.6%)	4^b^ (2.7%)	0.38 (0.05 to 2.81) *p* = 0.34	0
Mortality	3 RCTs 4 OS	[26, 27, 36, 37, 47, 48, 51]	RR	3104	1099	764^b^ (24.6%)	269^b^ (24.5%)	1.04 (0.85 to 1.28) *p* = 0.72	43

Statistically significant differences (*p* < 0.05) are denoted as bold

*OM*, Outcome measurement; *EMF*, Extramedullary fixation; *IMF*, Intramedullary fixation; *TAD*, Tip-apex distance; *RCT*, Randomized controlled trial; *OS*, Observational studies; *RR*, Relative risk; *MD*, Mean difference; *N.A.*, Not available

^a^Subgroup mean

^b^Cases reported in subgroup

### Surgical outcomes and operation characteristics

#### Mean time to bone healing (weeks)

**Fig. 13:**
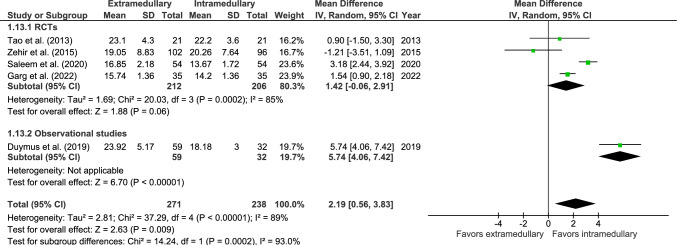
Forest plot of mean time to bone healing (weeks) after extramedullary versus intramedullary fixation of AO 31-A2 fractures

Mean time to radiological bone healing was reported in five studies: four RCTs [34, 37, 41, 42] and one observational study (Fig. 13) [46], with 271 patients treated with EM devices and 238 patients with IM devices. Mean time to bone healing was 19.6 weeks for EM devices and 17.8 weeks for IM devices. Bone healing time was significantly longer in EM fixation (MD 2.19 weeks, 95% CI 0.56–3.83, *p* = 0.009, *I*^2^ = 89%).

#### Poor radiological quality of reduction

Poor radiological quality of reduction using the classification by was reported in three studies: one RCT [41] and two observational studies (OR4, Figure S8) [46, 48]. Poor quality of reduction was reported in 23 out of 255 (9.0%) patients treated with an EM device and in 7 out of 253 (2.8%) patients treated with an IM. Tao et al. [41] reported zero cases. There was no significant difference between fixation groups (RR 2.52, 95% CI 0.71–8.93, *p* = 0.15, *I*^2^ = 56%).

#### Operation time (min)

**Fig. 14:**
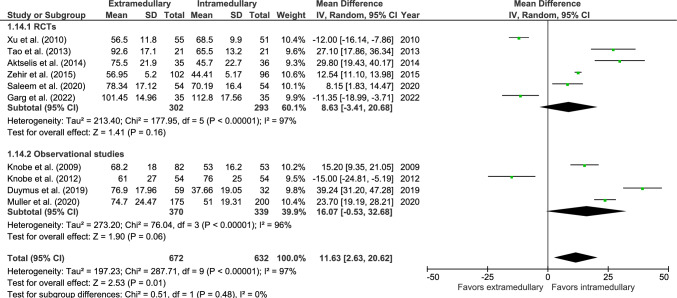
Forest plot of surgery duration (min) in extramedullary versus intramedullary fixation of AO 31-A2 fractures. Standard deviations for Knobe et al. [27] were imputed

Operation time was reported in 10 studies: six RCTs [32, 34, 36, 37, 41, 42] and four observational studies (Fig. 14) [27, 46–48], with 672 patients treated with EM devices and 632 patients treated with IM devices. The mean surgery duration was 71 min for EM fixation and 58 min for IM fixation. Operation time was significantly longer for EM fixation (MD 11.63 min, 95% CI 2.63–20.62, *p* = 0.01, *I*^2^ = 97%).

#### Hospital stay (days)

Hospital stay was reported in eight studies: three RCTs [36, 41, 42] and five observational studies (OR4, Figure S9) [27, 46–49], with 748 patients treated with EM devices and 549 patients treated with IM devices. The mean hospital stay was 11 days in EM fixation and 12 days in IM fixation. There was no significant difference between fixation groups (MD 0.63, 95% CI − 0.36–1.62, *p* = 0.21, *I*^2^ = 68%).

#### Blood loss and transfusion

The mean blood loss was 312 mL for EM fixation and 150 mL for IM fixation [34, 36, 37, 41, 42]. Blood loss was significantly higher in EM fixation (MD 134.5 mL, 95% CI 51.00–217.95, *p* < 0.001, *I*^2^ = 98%, OR4, Figure S10). There was no significant difference in transfusion rate [26, 27, 36, 47] (RR 1.09, 95% CI 0.68–1.74, *p* = 0.72, *I*^2^ = 87%, OR4 Figure S11) or number of number of blood replacement units [27, 46–48] (MD 0.23 units, 95% CI − 0.89–1.35, *p* = 0.69, *I*^2^ = 97%, OR4, Figure S12).

#### Fluoroscopy time (sec)

Fluoroscopy time was reported in five studies: three RCTs [36, 37, 41] and two observational study (OR4, Figure S13) [27, 47], with 314 patients treated with EM fixation and 275 patients treated with IM fixation. The mean fluoroscopy time was 122 s for EM fixation and 166 s for IM fixation. There was no significant difference between fixation groups (MD − 47.32 s, 95% CI − 142.63–47.99, *p* = 0.33, *I*^2^ = 99%).

#### Tip-apex distance (TAD) (mm) and TAD > 25mm

The mean TAD was 23 mm in EM fixation and 21 mm in IM fixation (OR4, Figure S14) [29, 33, 37, 47, 48]. There was no significant difference between fixation groups (MD 1.19 mm, 95% CI − 1.06–3.45, *p* = 0.30, *I*^2^ = 77%). Increased TAD > 25 mm was reported in two studies (OR4, Figure S15), with 60 cases out of 285 (21.1%) patients treated with an EM device and in 37 cases out of 300 (12.3%) patients treated with an IM device [26, 48]. The rate of TAD larger than 25 mm was significantly lower in the IM group (RR 1.73, 95% CI 1.10–2.74, *p* = 0.02, *I*^2^ = 18%).

#### Femoral neck shortening (mm)

Femoral neck shortening was reported in two studies: one RCT [28] and one observational study (OR4, Figure S16) [47], with 134 patients in the EM fixation group and 141 patients in the IM fixation group. The mean shortening distance was 8.0 mm for EM fixation and 3.1 mm for IM fixation. There was no significant difference between fixation groups (MD 4.00 mm, 95% CI − 3.84–11.84, *p* = 0.32, *I*^2^ = 96%).

#### Neck-shaft angle (NSA) (°)

NSA was reported in two observational studies (OR 4, Figure S17) [42, 46, 47]. The extramedullary fixation group consisted of 129 patients with a mean of 129.9° and the intramedullary fixation group consisted of 106 patients with a mean of 123.7°. There was no difference between fixation groups (MD 4.67, 95% CI − 2.58–11.92, *p* = 0.21, *I*^2^ = 90%).

### Costs

#### Costs- and cost-effectiveness

No comparable data on costs- or cost-effectiveness could be extracted from any of the included articles.

An overview of all surgical outcomes is given in Table 4.

**Table 4:** Overview of surgical outcomes and operation characteristics

Outcome	Study type	References	OM	Total population EMF	Total population IMF	Mean/cases		Pooled effect (95% CI), *p*-value	*I*^2^ (%)
						EMF	IMF		
Mean time to bone healing (weeks)	4 RCTs 1 OS	[34, 37, 41, 46]	MD	271	238	19.6^a^	17.8^a^	2.19 (0.56 to 3.83) ***p* = 0.009**	89
Poor radiological quality of reduction	1 RCT 2 OS	[41, 46, 48]	RR	255	253	23^b^ (9.0%)	7^b^ (2.8%)	2.52 (0.71 to 8.93) *p* = 0.15	56
Surgery duration (min)	6 RCTs 4 OS	[27, 32, 34, 36, 37, 41, 46–48]	MD	672	632	71^a^	58^a^	11.63 (2.63 to 20.62) ***p* = 0.01**	97
Hospital stay (days)	3 RCTs 5 OS	[27, 36, 41, 46–49]	MD	748	549	11^a^	12^a^	0.63 (− 0.36 to 1.62) *p* = 0.21	68
Blood loss (mL)	5 RCTs	[34, 36, 37, 41]	MD	267	257	312^a^	150^a^	134.5 (51.0 to 218) ***p* = 0.002**	98
Patients receiving blood transfusion	2 RCTs 2 OS	[26, 27, 36, 47]	RR	301	258	162^b^ (53.8%)	129^b^ (50.0%)	1.09 (0.68 to 1.74) *p* = 0.72	87
Blood replacement units	4 OS	[27, 46–48]	MD	288	286	1.69^a^	1.3^a^	0.54 (0.67 to 1.75) *p* = 0.38	97
Fluoroscopy time (sec)	3 RCTs 2 OS	[27, 36, 37, 41, 47]	MD	314	275	122^a^	166^a^	− 47.32 (− 142.63 to 47.99) *p* = 0.33	99
TAD (mm)	2 RCTs 3 OS	[29, 33, 37, 47, 48]	MD	404	428	23^a^	21^a^	1.19 (− 1.06 to 3.45) *p* = 0.30	77
TAD > 25 mm	1 RCT 1 OS	[26, 48]	RR	285	300	60^b^ (21.1%)	37^b^ (12.3%)	1.73 (1.10 to 2.74) ***p* = 0.02**	18
Femoral neck shortening (mm)	1 RCT 1 OS	[28, 47]	MD	134	141	8.0^a^	3.1^a^	4.00 (− 3.84 to 11.84) *p* = 0.32	96
NSA (°)	1 RCT 2 OS	[42, 46, 47]	MD	129	106	129.9^a^	123.7^a^	4.67 (− 2.58 to 11.92) *p* = 0.21	90

*OM*, Outcome measurement; *EMF*, Extramedullary fixation; *IMF*, Intramedullary fixation; *TAD*, Tip-apex distance; *NSA*, neck-shaft angle; *RCT*, Randomized controlled trial; *OS*, Observational studies; *RR*, Relative risk; *MD*, Mean difference

^a^Subgroup mean

^b^Cases reported in subgroup

## Discussion

This systematic review and meta-analysis compared functional outcomes, complications, and surgical outcomes for EM versus IM fixation in elderly patients with an AO type 31-A2 fracture. Statistically superior results in favor of IM fixation were found for several outcomes including Harris Hip Score, Parker mobility score, lower extremity measure, time to full weight bearing, superficial infection, nonunion, fixation failure, leg shortening, time to bone healing, and surgery duration.

The most recent Cochrane review, by Lewis et al. [53], on RCTs and ‘RCT-like’ cohort studies published up to July 2020 compared EM and IM fixation for a combination of AO A1, A2, and A3 fractures. In correspondence with the current review it found an increased risk of several complications including nonunion and implant failure in patients treated with an EM device. However, it found no difference in functional outcomes and found that IM devices were associated with an increased intra- and postoperative peri-implant fracture and shorter HLOS. Differences found in comparison with this meta-analysis can be partly explained by its combination of A1, A2, and A3 fractures and inclusion of older studies (before 2005) with a relatively higher rate of complications. The Cochrane review only performed a stratified analysis for stable versus unstable fractures for reoperation (no significant difference) and did not assess surgical outcomes and operation characteristics.

Another recent meta-analysis by Wessels et al*.* [54] comparing IM nailing with sliding hip screws (SHS) for all combined AO 31-A fractures reported no significant differences between both fixation options for the 31-A2 subgroup in combined major complication rate, infections (superficial and deep infections combined), nonunion, and mortality. Wessels et al*.* included several articles also included in this analysis, but chose to combine major complications, while the current study explores a wider range of adverse events, biomechanical outcomes, and patient-reported outcomes. Contrary to the current study, they did not find a significant difference for nonunion rate. This review includes nonunion rate from two more recent RCT’s not included by Wessels et al*.*, shifting the effect toward IM fixation. A meta-analysis of RCT’s published by Zhu et al*.* [55], compared IM nails with SHS for AO 31-A2 fractures. They also showed statistical superiority of IM nails for intraoperative blood loss, leg shortening, superficial infections, length of hospital stay, days to mobilization, and the Parker mobility score. These results are similar to those found in this meta-analysis.

Results found by older reviews and meta-analyses, demonstrating inferiority of IM fixation based on older studies, featuring mainly first and second generation IM implants, should be considered obsolete nowadays [16, 56]. A change in paradigm that was already predicted by Bhandari et al. [57]. Future meta-analyses should only incorporate implants that are still clinically used.

### Interpretation of results

Several points should be considered when interpreting the differences found between EM and IM fixation. In contrast to the meta-analysis of several of the major complications (e.g., to prosthesis, mortality and infections) and their sequelae (e.g., reoperation, conversion to prosthesis), a relative lack of data on functional outcomes in both the number of (prospective) studies and the number of included patients was observed. This study showed a significant mean difference of 4.1 points on the Harris Hip Score, on a scale of 0–100 points, in favor of intramedullary fixation. This number should be considered in the light of the minimally clinical important difference, which is established at 15 points for the HHS [52]. While statistically significant, this difference is not expected to be clinically relevant. Similar arguments could be made for the small differences found in the Parker mobility score (MD − 0.67, on 0–9 scale) and LEM (MD − 4.07 on 0–100 scale), although no minimal important change/difference values have been published for these measures. The difference to full weight bearing (MD 1.04 weeks in favor of IM fixation) could in theory be clinically relevant. However, this outcome was reported in only two studies which used radiological union as a starting point for full weight bearing, making this outcome similar to the outcome radiological union mentioned in other studies (favoring IM fixation). Relevant would be a difference in patient-reported time to full weight bearing without restrictions given by the treating surgeon that exceeds the MICD.

With regard to complications and operation characteristics several considerations should be taken into account. Most fracture- and implant-related complications are rare and occur at rates under 5% or even 2%. In the two complications with the largest number of included patients, reoperation, and conversion to prosthesis, no significant differences were found. Although reoperation or conversion can be expected to be a result of other complications such as nonunion, infection, or fixation failure, statistically significant differences were found for these three complications. These effects might be exaggerated due to several (randomized) studies with 0 cases in both study arms, that could not be included in a pooled effect measure. The mean follow-up was 12 months; however, the rate of biomechanical complications increases with a longer follow-up time. Therefore, comparing studies with varying follow-up durations might have influenced the meta-analysis. Nearly all operation characteristics or surgical outcomes suffer from very high heterogeneity and variables such as surgery time or measurement of blood loss are often poorly defined by studies. In combination with their relatively low patient numbers and the possible influence of retrospective data, these results should be interpreted with care.

While this review demonstrates that IM fixation for AO 31-A2 trochanteric fractures is no longer inferior to EM fixation, superiority remains questionable. Many differences are below clinically relevant thresholds, hold low quality of evidence, or analyses are underpowered to adequately compare functional outcomes or rare complications. Where clear superiority is missing, costs-effectiveness should also be considered when selecting an EM or IM fixation approach. Currently, EM fixation is considered the most cost-effective approach by the few studies that were conducted on this topic [15, 26]. This conclusion is also emphasized by the Dutch and U.K. guidelines for treatment of proximal femoral fractures [13, 14]. This is mainly due to the generally higher IM device cost. However, extensive cost-effectiveness analyses, including broad health care and rehabilitation costs, quality of life, and functional outcomes do not exist at the time of writing. Such an analysis would provide critical data for updating current guidelines. Because of the shifting trend of effectiveness toward IM fixation, due to newer implants and operative strategies, these conclusions may have become outdated. Therefore, this study reaffirms the need for properly powered, large-scale comparisons of both fixation strategies, including costs and costs-effectivity. Additionally, due to the relative rarity of major complications and relative lack of functional or patient-reported data, future research should primarily focus on functional outcomes and quality of life, instead of primarily focusing on number of complications.

### Limitations

This is the most extensive systematic review and meta-analysis that is restricted to the treatment of only AO 31-A2 proximal femoral fractures, including both observational and RCT data on currently available implants, to date. It includes a substantially larger population than all previous meta-analyses. However, the meta-analysis does have several limitations: There was high heterogeneity for many analyzed variables due to differences in studied implants, study designs, international differences, and duration of studies. In addition to this, many studies can be considered underpowered for often rare outcomes. Large (observational) studies can strongly impact the overall effect. This, together with a high heterogeneity could have resulted in missing or overstated differences between IM and EM fixation. Multiple functional scores and pain measurements were used by the included studies and SDs were often not reported. This made calculation of pooled effects impossible for several functional outcome scores. In addition, not all studies reporting functional scores also included baseline scores, making it difficult to observe potential selection bias. There are sparse data on many of the included variables, as many studies included all three of the subtypes of trochanteric fractures and were only adequately powered for the complete population. While the AO type A2 fracture subgroups could be extracted and included in the meta-analysis, this could result in a lower generalizability and lower quality of evidence for these limited results. Lastly, data for all specific outcomes were included as described by the original articles. Most studies did not provide extensive definitions or definitions of outcomes differed slightly between studies. While articles were reviewed extensively and only comparable data were included, this might have introduced bias, especially in the case of retrospective data.

## Conclusion

This review and meta-analysis showed that several functional outcomes, complications, and surgical outcomes were in favor of intramedullary fixation when compared with extramedullary fixation of AO type 31-A2 fractures. Results indicate significantly higher Harris hip score, Parker mobility score, lower extremity measure, and recovery to pre-operative walking ability. No difference was found in reoperation rate or conversion to prosthesis, but studies show a decrease in superficial infections, nonunion, fixation failure, leg shortening, surgery duration, operative blood loss, and increased tip-apex distance, all in favor of intramedullary fixation. Previous meta-analyses describing inferiority of IM fixation in AO type 31-A2 fractures should now be considered obsolete. However, a true superiority of IM fixation for AO type 31-A2 fractures remains questionable as several differences in functional outcomes appear not clinically relevant, data on many outcomes remains sparse or heterogeneous, and a detailed cost(-effectiveness) evaluation of modern IM nails is missing in the literature. As major complications are rare and there is a relative lack of functional, patient-reported, and cost data, future research should primarily focus on functional outcomes, quality of life, and costs-effectiveness, instead of primarily focusing on the number of complications.

### Supplementary Information

Below is the link to the electronic supplementary material.

Supplementary file1 (DOCX 17 KB)

Supplementary file2 (DOCX 16 KB)

Supplementary file3 (DOCX 158 KB)

Supplementary file4 (DOCX 351 KB)

## Data Availability

The data that support the findings of this study are available from the corresponding author, upon reasonable request.
