# Supplementary material for: Extramedullary versus intramedullary fixation of unstable trochanteric femoral fractures (AO type 31-A2): a systematic review and meta-analysis
Source: Arch Orthop Trauma Surg. 2024 Jan 4;144(3):1189–209. doi: 10.1007/s00402-023-05138-9 (PMC10896832; doi:10.1007/s00402-023-05138-9)
Supplement: Supplementary file 1 — Supplementary file1 (DOCX 17 KB) [file 402_2023_5138_MOESM1_ESM.docx]

**Online resource 1: Search strategy**

**Table S1: Results of initial search (22 March 2021)**

| **Database searched** | **Platform** | **References** | **After de-duplication** |
| --- | --- | --- | --- |
| Embase | Embase.com | 5,107 | 5,023 |
| Medline ALL | Ovid | 4,037 | 536 |
| Web of Science Core Collection* | Web of Knowledge | 2,807 | 432 |
| Cochrane Central Register of Controlled Trials | Wiley | 538 | 126 |
| **Subtotal** |  | **12,489** | **6,117** |
| Other sources: Google Scholar (200 top-ranked) | | 200 | 39 |
| **Total** | | **12,689** | **6,156** |

*Science Citation Index Expanded (1975-present); Social Sciences Citation Index (1975-present); Arts & Humanities Citation Index (1975-present); Conference Proceedings Citation Index- Science (1990-present); Conference Proceedings Citation Index- Social Science & Humanities (1990-present); Emerging Sources Citation Index (2015-present)

**Table S2: Results of search update (26 September 2022)**

| **Database searched** | **Platform** | **Years of coverage** | **Records** | **Records after duplicates removed** |
| --- | --- | --- | --- | --- |
| Medline ALL | Ovid | 1946 - Present | 4525 | 4490 |
| Embase | Embase.com | 1971 - Present | 5720 | 1766 |
| Web of Science Core Collection* | Web of Knowledge | 1975 - Present | 3548 | 573 |
| Cochrane Central Register of Controlled Trials** | Wiley | 1992 - Present | 584 | 107 |
| Additional Search Engines: Google Scholar (200 top-ranked) | | | 200 | 40 |
| **Total** | | | **14577** | **6976** |

*Science Citation Index Expanded (1975-present); Social Sciences Citation Index (1975-present); Arts & Humanities Citation Index (1975-present); Conference Proceedings Citation Index- Science (1990-present); Conference Proceedings Citation Index- Social Science & Humanities (1990-present); Emerging Sources Citation Index (2005-present)** Manually deleted abstracts from trial registries. No other database limits were used than those specified in the search strategies

Search terms used in both initial search and update:

**Embase**

('femur intertrochanteric fracture'/exp OR 'femur pertrochanteric fracture'/exp OR 'femur trochanteric fracture'/exp OR 'proximal femur fracture'/de OR (((trochanter* OR pertrochanter* OR intertrochanter*) NEAR/6 (fractur*)) OR ((prox*-femur* OR prox*-femoral*) NEAR/3 (fract*)) OR 31A OR 31A1 OR 31A2 OR 31A3 OR 31-A1 OR 31-A2 OR 31-A3):ab,ti,kw) **AND** ('intramedullary nailing'/exp OR 'intramedullary nail'/exp OR 'bone nail'/exp OR 'bone pin'/exp OR 'bone plate'/exp OR 'bone screw'/exp OR 'plate fixation'/exp OR 'internal fixator'/de OR 'osteosynthesis'/exp OR (CMN OR EPFN OR PFNA OR PFN OR IMN OR BCM OR DLT OR SHS OR DHS OR PCCP OR PFLP OR TFNA OR intramedullar* OR extramedullar* OR intra-medullar* OR extra-medullar* OR cephalomedullar* OR cephalomedullar* OR nail* OR plate* OR plating OR screw* OR pin* OR rod* OR nagel* OR dyna-locking* OR antigrad* OR ((internal*) NEAR/3 (fixat*)) OR osteosynthes* OR osteo-synthes*):ab,ti,kw) NOT ([Conference Abstract]/lim)

**Medline**

((((trochanter* OR pertrochanter* OR intertrochanter*) ADJ6 (fractur*)) OR ((prox*-femur* OR prox*-femoral*) ADJ3 (fract*)) OR 31A OR 31A1 OR 31A2 OR 31A3 OR 31-A1 OR 31-A2 OR 31-A3).ab,ti,kf.) AND (exp Fracture Fixation, Internal/ OR Internal Fixators OR Bone Nails/ OR Bone Plates/ OR exp Bone Screws/ OR (CMN OR EPFN OR PFNA OR PFN OR IMN OR BCM OR DLT OR SHS OR DHS OR PCCP OR PFLP OR TFNA OR intramedullar* OR extramedullar* OR intra-medullar* OR extra-medullar* OR cephalomedullar* OR cephalomedullar* OR nail* OR plate* OR plating OR screw* OR pin* OR rod* OR nagel* OR dyna-locking* OR antigrad* OR ((internal*) ADJ3 (fixat*)) OR osteosynthes* OR osteo-synthes*).ab,ti,kf.) NOT (news OR congres* OR abstract* OR book* OR chapter* OR dissertation abstract*).pt.

**Cochrane**

((((trochanter* OR pertrochanter* OR intertrochanter*) NEAR/6 (fractur*)) OR (((prox* NEXT/1 femur*) OR (prox* NEXT/1 femoral*)) NEAR/3 (fract*)) OR 31A OR 31A1 OR 31A2 OR 31A3 OR (31 NEXT/1 A1) OR (31 NEXT/1 A2) OR (31 NEXT/1 A3)):ab,ti,kw) **AND** ((CMN OR EPFN OR PFNA OR PFN OR IMN OR BCM OR DLT OR SHS OR DHS OR PCCP OR PFLP OR TFNA OR intramedullar* OR extramedullar* OR (intra NEXT/1 medullar*) OR (extra NEXT/1 medullar*) OR cephalomedullar* OR cephalomedullar* OR nail* OR plate* OR plating OR screw* OR pin* OR rod* OR nagel* OR (dyna NEXT/1 locking*) OR antigrad* OR ((internal*) NEAR/3 (fixat*)) OR osteosynthes* OR (osteo NEXT/1 synthes*)):ab,ti,kw)

**Web of Science**

TS=(((((trochanter* OR pertrochanter* OR intertrochanter*) NEAR/5 (fractur*)) OR (((prox* NEAR/1 femur*) OR (prox* NEAR/1 femoral*)) NEAR/2 (fract*)) OR 31A OR 31A1 OR 31A2 OR 31A3 OR 31-A1 OR 31-A2 OR 31-A3)) AND ((CMN OR EPFN OR PFNA OR PFN OR IMN OR BCM OR DLT OR SHS OR DHS OR PCCP OR PFLP OR TFNA OR intramedullar* OR extramedullar* OR intra-medullar* OR extra-medullar* OR cephalomedullar* OR cephalomedullar* OR nail* OR plate* OR plating OR screw* OR pin* OR rod* OR nagel* OR dyna-locking* OR antigrad* OR ((internal*) NEAR/2 (fixat*)) OR osteosynthes* OR osteo-synthes*))) AND DT=(Article OR Review OR Letter OR Early Access)

**Google Scholar**

"intertrochanteric|pertrochanteric|trochanteric fracture" intramedullar|extramedullar

**PubMed**

((((trochanter*[tiab] OR pertrochanter*[tiab] OR intertrochanter*[tiab]) AND (fractur*[tiab])) OR ((prox*[tiab]-femur*[tiab] OR prox*[tiab]-femoral*[tiab]) AND (fract*[tiab])) OR 31A OR 31A1 OR 31A2 OR 31A3 OR 31-A1 OR 31-A2 OR 31-A3)) AND (Fracture Fixation, Internal[mh] OR Internal Fixators OR Bone Nails[mh] OR Bone Plates[mh] OR Bone Screws[mh] OR (CMN OR EPFN OR PFNA OR PFN OR IMN OR BCM OR DLT OR SHS OR DHS OR PCCP OR PFLP OR TFNA OR intramedullar*[tiab] OR extramedullar*[tiab] OR intra-medullar*[tiab] OR extra-medullar*[tiab] OR cephalomedullar*[tiab] OR cephalomedullar*[tiab] OR nail*[tiab] OR plate*[tiab] OR plating OR screw*[tiab] OR pin*[tiab] OR rod*[tiab] OR nagel*[tiab] OR dyna-locking*[tiab] OR antigrad*[tiab] OR ((internal*[tiab]) AND (fixat*[tiab])) OR osteosynthes*[tiab] OR osteo-synthes*[tiab])) NOT (news[pt] OR congres*[tiab] OR abstract*[tiab] OR book*[tiab] OR chapter*[tiab] OR dissertation abstract*[tiab])

**Extramedullary versus intramedullary fixation of unstable trochanteric femoral fractures (AO type 31-A2): a systematic review and meta-analysis**

Archives of Orthopaedic and Trauma Surgery

Miliaan L. Zeelenberg^1#^, MD; A. Cornelis Plaisier^1#^, BSc; Leendert H.T. Nugteren^1^, BSc; Sverre A.I. Loggers^1,2^, MD; Pieter Joosse^2^, MD PhD; Michiel H.J. Verhofstad^1^, MD PhD; Dennis Den Hartog^1^, MD PhD; Esther M.M. Van Lieshout^1^, PhD MSc; STABLE-HIP Study Group*

^1^ Trauma Research Unit Department of Surgery, Erasmus MC, University Medical Center Rotterdam, Rotterdam, The Netherlands

^2^ Department of Surgery, Noordwest Ziekenhuisgroep, Alkmaar, The Netherlands

^#^ Both first authors contributed equally

*Taco Gosens, MD PhD; Johannes H. Hegeman, MD PhD; Suzanne Polinder; Rudolf W. Poolman, MD PhD; Hanna C. Willems; Rutger G. Zuurmond

**Corresponding authors**

Dr. E.M.M. Van Lieshout

Trauma Research Unit Department of Surgery

Erasmus MC, University Medical Center Rotterdam

P.O. Box 2040

3000 CA Rotterdam

The Netherlands

Phone: +31.10.7031050

Mail: [e.vanlieshout@erasmusmc.nl](mailto:e.vanlieshout@erasmusmc.nl)
