# Supplementary material for: Extramedullary versus intramedullary fixation of unstable trochanteric femoral fractures (AO type 31-A2): a systematic review and meta-analysis
Source: Arch Orthop Trauma Surg. 2024 Jan 4;144(3):1189–209. doi: 10.1007/s00402-023-05138-9 (PMC10896832; doi:10.1007/s00402-023-05138-9)
Supplement: Supplementary file 2 — Supplementary file2 (DOCX 16 KB) [file 402_2023_5138_MOESM2_ESM.docx]

**Online resource 2: Devices used in AO type 31-A2 fracture fixation**

**Supplemental Table S3: Extramedullary and intramedullary devices used per study (as described in article)**

| **Study** | **Extramedullary device** | **Intramedullary device** |
| --- | --- | --- |
| Aktselis *et al.* (2014) (31) | AMBI sliding hip screw^1^ (SHS) | (Short) Gamma Nail^2^ (GN) |
| Andalib *et al.* (2020) (32) | Dynamic hip screw (DHS) or dynamic condylar screw (DCS)^*^ | Cephalomedullary nail^*^ (CMN) |
| Andruszkow *et al.* (2012) (42) | Dynamic hip screw^3^ (DHS) | Gamma 3 nail^2^ (GN) |
| Barton *et al.* (2010) (25) | Sliding hip screw (SHS)^*^ | Gamma nail (GN)^*^ |
| Butt *et al.* (2017) (43) | Dynamic hip screw (DHS) with or without locking plate^*^ | Intramedullary nail (IMN)^*^ |
| Crespo *et al.* (2012) (44) | Percutaneus compression plate^4^ (PCCP) | Gamma nail^2^ (GN) |
| Duymus *et al.* (2019) (45) | Dynamic hip screw^3^ (DHS) or Proximal femoral locking compression plate (PF-LCP)^3^ | Trigen Intertan^2^ |
| Garg *et al.* (2022) (41) | Dynamic hip screw^3^ (DHS) | Proximal femoral nail^3^ (PFN) |
| Grønhaug *et al.* (2022) (52) | SHS with or without a trochanteric support plate (TSP)^*^ | Intramedullary nail (IMN) short or long^*^ |
| Knobe *et al.* (2009) (26) | Percutaneus compression plate^4^ (PCCP) or Dynamic hip screw^5^ (DHS) | Proximal femoral nail^5^ (PFN) |
| Knobe *et al.* (2012) (46) | Percutaneus compression plate^4^ (PCCP) | Proximal femoral nail antirotation^3^ (PFNA) |
| Müller *et al.* (2020) (47) | Dynamic hip screw^3^ (DHS) with or without trochanteric fixation plate (TFP) | Proximal femoral nail antirotation^3^ (PFNA) |
| Ovesen *et al.* (2006) (37) | Dynamic hip screw^3^ (DHS), with or without stabilizing plate | Trochanteric gamma nail (GN)^2^, second generation |
| Page *et al.* (2016) (48) | Dynamic hip screw^3^ (DHS) | Intramedullary nail^*^ (IMN) |
| Pajarinen *et al.* (2005) (38) | Dynamic hip screw^3^ (DHS) | Proximal femoral nail^3^ (PFN) |
| Parker *et al*. (2017) (39) | Sliding hip screw^*^ (SHS) | Targon proximal femoral (TPF) nail^7^ + Targon proximal femoral telescrew (TPFT) nail^7^ |
| Pyrhönen *et al.* (2022) (51) | Sliding hip screw^*^ (SHS) | Intramedullary nail^*^ (IMN), short and long |
| Reindl *et al.* (2015) (27) | Dynamic hip screw^3^ (DHS) | Trochanteric fixation nail^3^ (TFN) or Gamma nail^2^ (GN), or Trigen INTERTAN nail^1^ |
| Saleem *et al.* (2020) (33) | Dynamic hip screw^3^ (DHS) | Proximal femoral nail^3^ (PFN) |
| Sevinç *et al.* (2020) (28) | Dynamic hip screw^3^ (DHS) | Proximal femoral nail antirotation^3^ (PFNA) |
| Singh *et al.* (2017) (34) | Proximal femoral locking compression plate (PFLCP)^*^ | Proximal femoral nail^3^ (PFN) |
| Suh *et al.* (2015) (49) | Compression hip screw^8^ (CHS) | Proximal femoral nail antirotation^3^ (PFNA) |
| Tao *et al.* (2013) (40) | Reverse less invasive stabilization system (LISS)^3^ | Proximal femoral nail antirotation^3^ (PFNA) |
| Tucker *et al.* (2018) (50) | Sliding hip screws^3,9^ (SHS) | Cephalomedullary nails^1,3^ (CMN) |
| Verettas *et al*. (2010) (29) | Dynamic hip screw^3^ (DHS) | Gamma nail^2^ (GN) or Endovis BA^6^ (EBA) |
| Xu *et al*. (2010) (35) | Dynamic hip screw^3^ (DHS) | Proximal femoral nail antirotation^3^ (PFNA) |
| Zehir *et al.* (2015) (36) | Dynamic hip screw^3^ (DHS) | Proximal femoral nail antirotation^3^ (PFNA) |

**^1^** Smith & Nephew, Memphis, TN, USA

^2^ Stryker, Schönkirchen, Germany/Mahwha, New Jersey, USA

^3^ Depuy-Synthes, Westchester, PA,USA/Umkirch, Germany/Oberdorf, Switserland

^4^ Orthofix, Bussolengo, Italy/Mckinney, TX, USA

^5^ Clinical house GmBh, Bochum, Germany

^6^ Citieffe, Bolongna, Italy

^7^ B. Braun, Tuttlingen, Germany

^8^ Corentec, Seoul, Korea

^9^ AO, Dubendorf, Switserland

^*^ Device manufacturer not mentioned in article

**Extramedullary versus intramedullary fixation of unstable trochanteric femoral fractures (AO type 31-A2): a systematic review and meta-analysis**

Archives of Orthopaedic and Trauma Surgery

Miliaan L. Zeelenberg^1#^, MD; A. Cornelis Plaisier^1#^, BSc; Leendert H.T. Nugteren^1^, BSc; Sverre A.I. Loggers^1,2^, MD; Pieter Joosse^2^, MD PhD; Michiel H.J. Verhofstad^1^, MD PhD; Dennis Den Hartog^1^, MD PhD; Esther M.M. Van Lieshout^1^, PhD MSc; STABLE-HIP Study Group*

^1^ Trauma Research Unit Department of Surgery, Erasmus MC, University Medical Center Rotterdam, Rotterdam, The Netherlands

^2^ Department of Surgery, Noordwest Ziekenhuisgroep, Alkmaar, The Netherlands

^#^ Both first authors contributed equally

*Taco Gosens, MD PhD; Johannes H. Hegeman, MD PhD; Suzanne Polinder; Rudolf W. Poolman, MD PhD; Hanna C. Willems; Rutger G. Zuurmond

**Corresponding authors**

Dr. E.M.M. Van Lieshout

Trauma Research Unit Department of Surgery

Erasmus MC, University Medical Center Rotterdam

P.O. Box 2040

3000 CA Rotterdam

The Netherlands

Phone: +31.10.7031050

Mail: [e.vanlieshout@erasmusmc.nl](mailto:e.vanlieshout@erasmusmc.nl)
