# Supplementary material for: Extramedullary versus intramedullary fixation of unstable trochanteric femoral fractures (AO type 31-A2): a systematic review and meta-analysis
Source: Arch Orthop Trauma Surg. 2024 Jan 4;144(3):1189–209. doi: 10.1007/s00402-023-05138-9 (PMC10896832; doi:10.1007/s00402-023-05138-9)
Supplement: Supplementary file 3 — Supplementary file3 (DOCX 158 KB) [file 402_2023_5138_MOESM3_ESM.docx]

**Online resource 3: Funnel plots for included outcomes**

**Supplemental Figure S1: Funnel plots for included outcomes after extramedullary versus intramedullary fixation of AO 31-A2 fractures**

| **A: Harris Hip Score** | **B: Parker mobility score** |
| --- | --- |
| **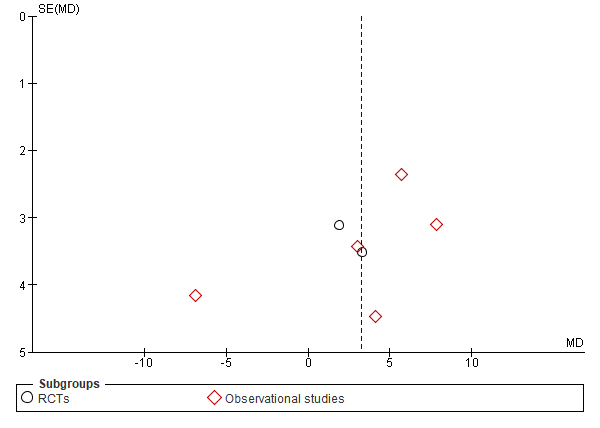** | **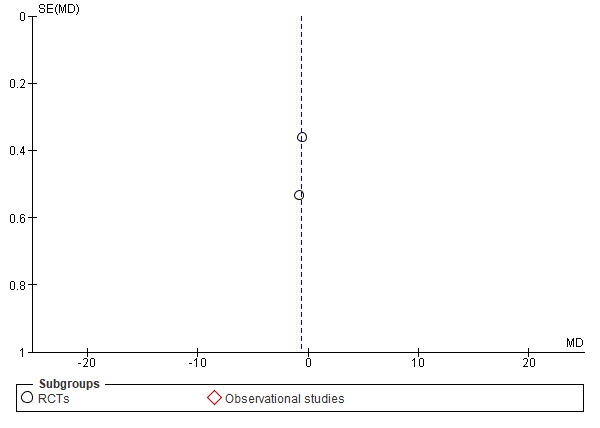** |
| **C: Lower extremity measure** | **D: Recovery to pre-operative walking ability** |
| **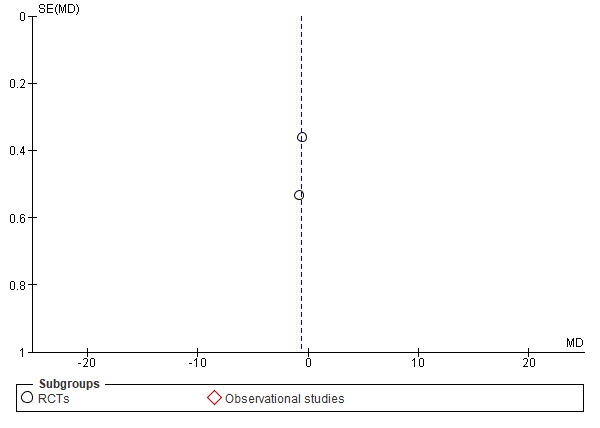** | **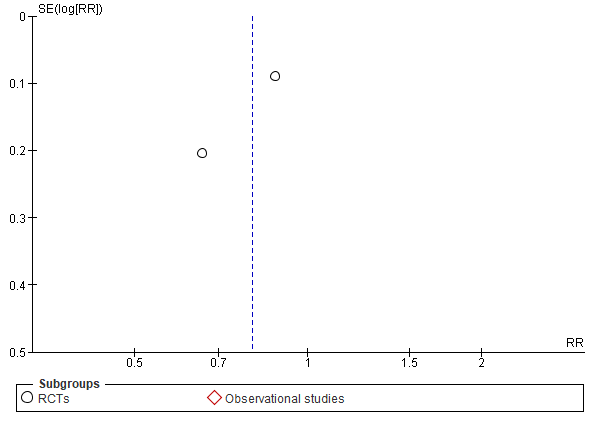** |
| **E: Reoperations** | **F: Deep infections** |
| **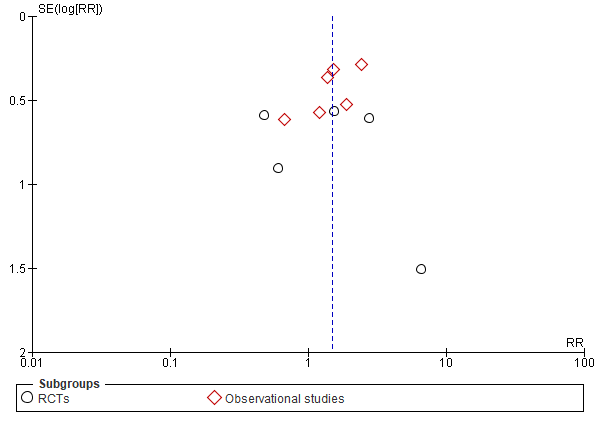** | **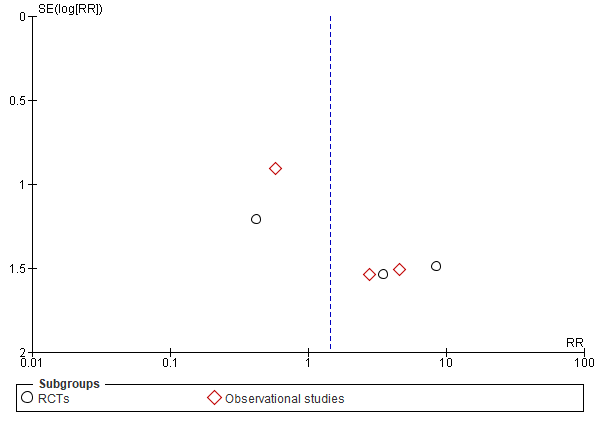** |
| **G: Superficial infections** | **H: Nonunion** |
| **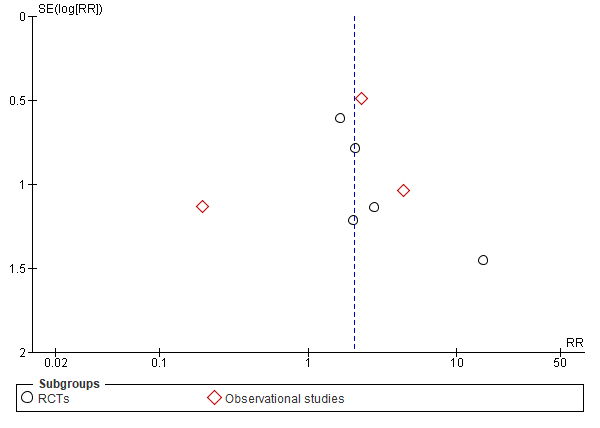** | **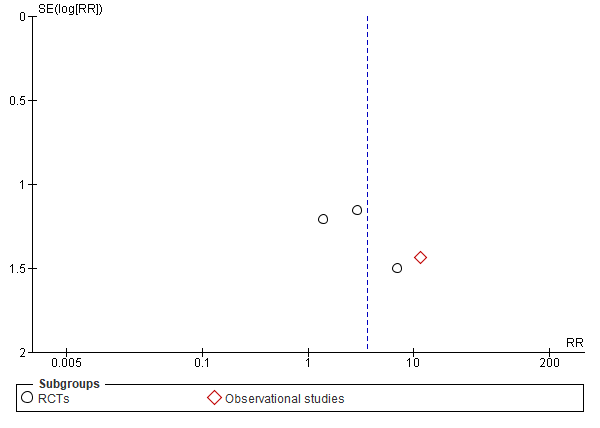** |
| **I: Cut-out** | **J: Peri-implant fracture** |
| **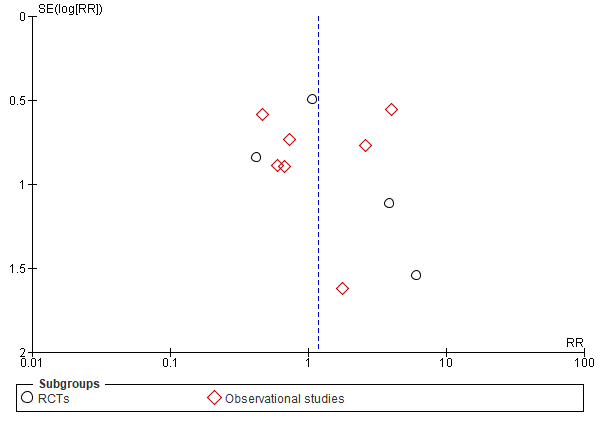** | **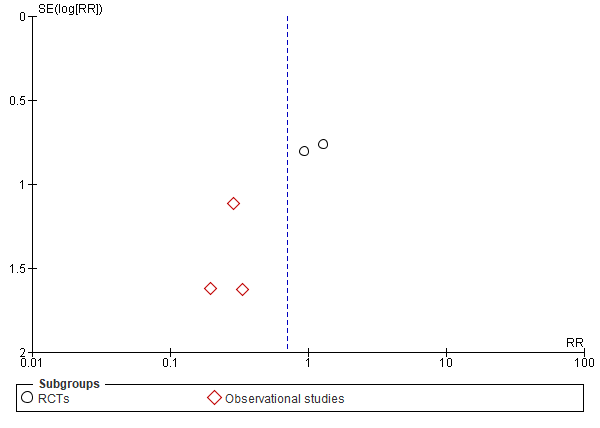** |
| **K: Conversion to prosthesis** | **L: Implant/fixation failure** |
| **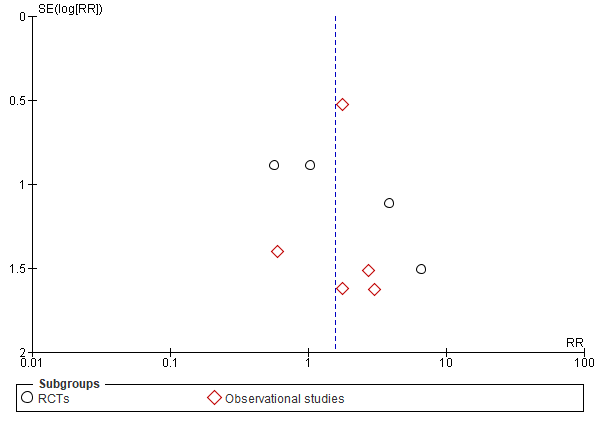** | **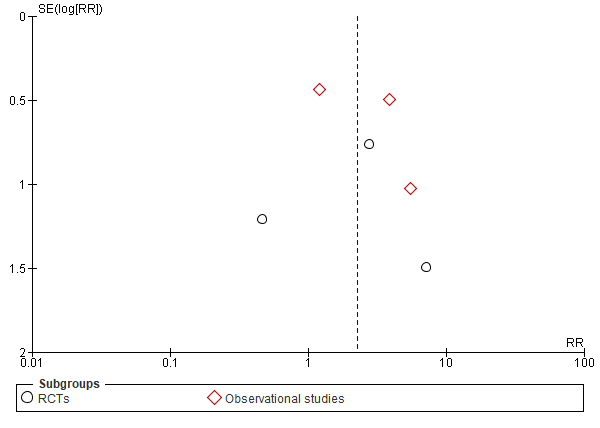** |
| **M: Leg shortening** | **N: Screw Migration** |
| **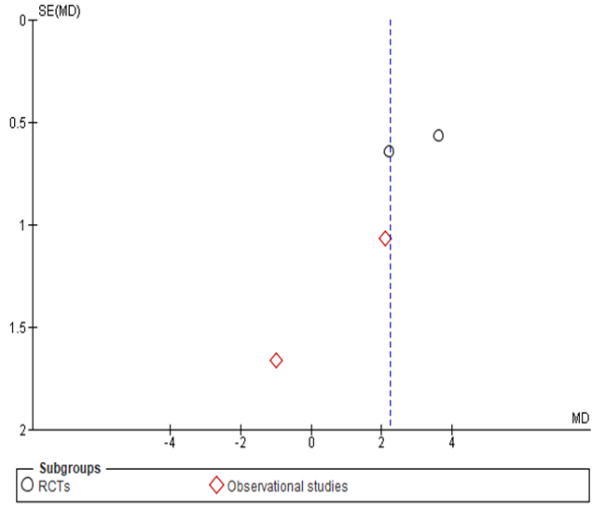** | **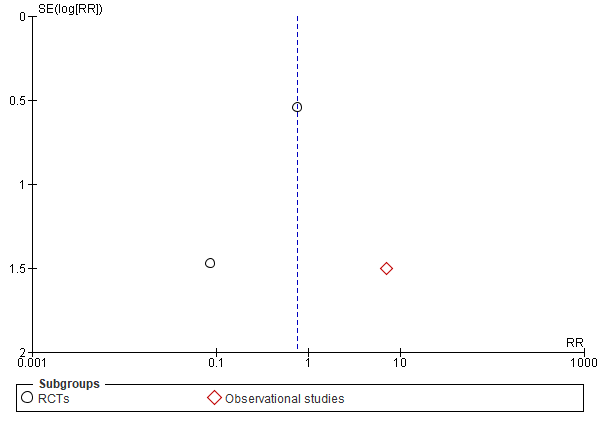** |
| **O: Femoral shaft fracture** | **P: Mortality** |
| **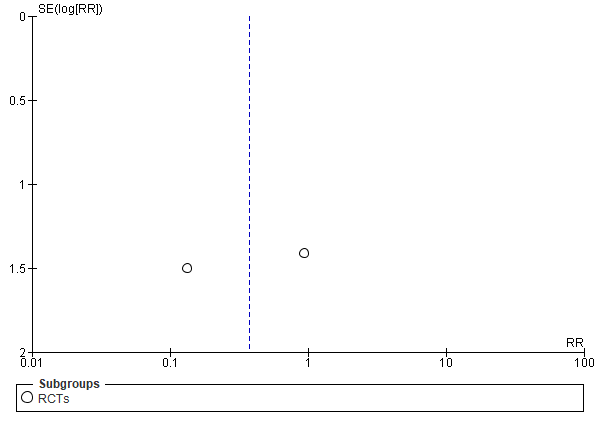** | **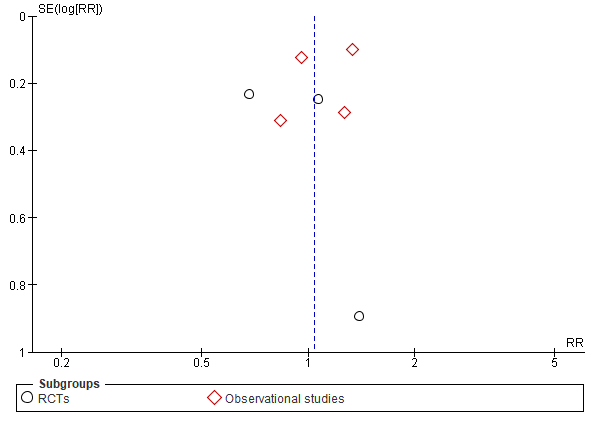** |
| **Q: Time to bone healing** | **R: Poor quality of reduction** |
| **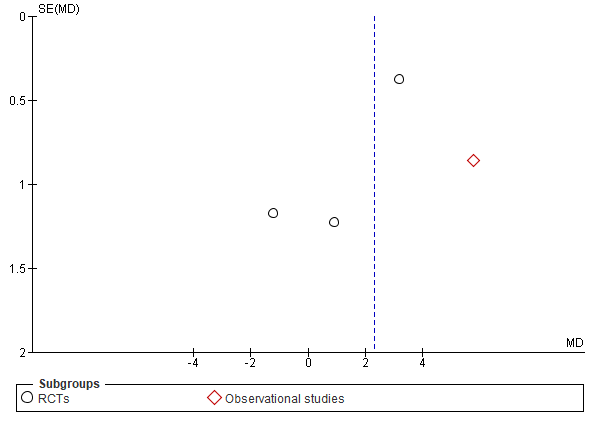** | **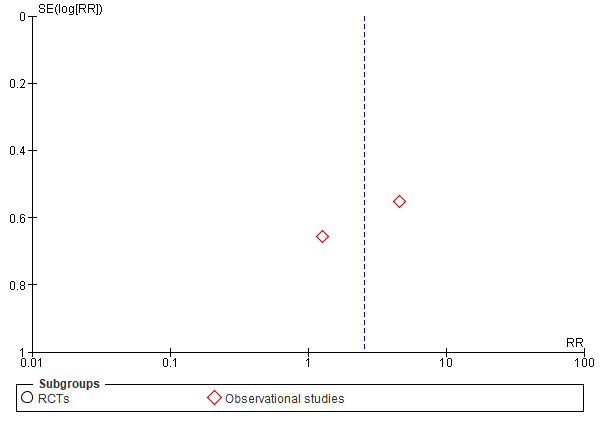** |
| **S: Surgery duration** | **T: Hospital stay** |
| **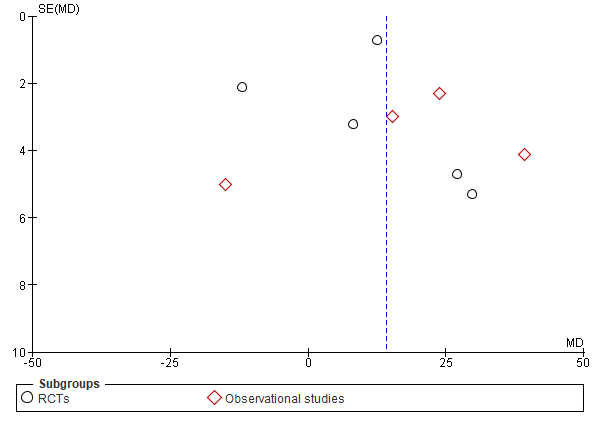** | **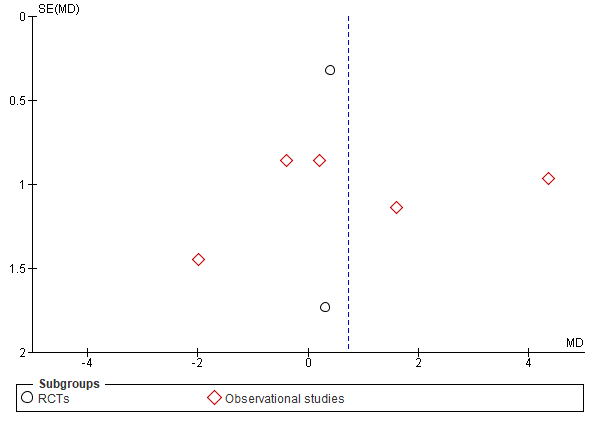** |
| **U: Blood loss** | **V: Blood replacement units** |
| **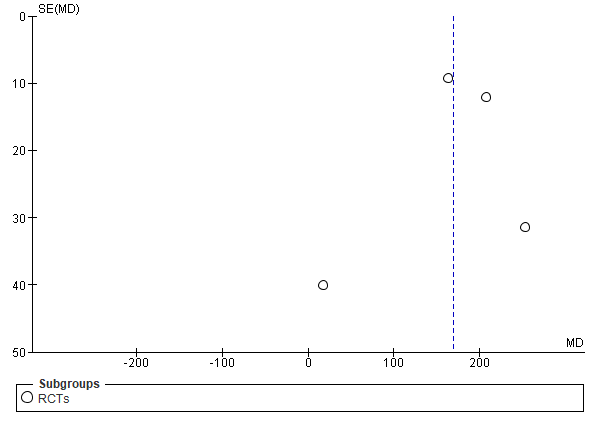** | **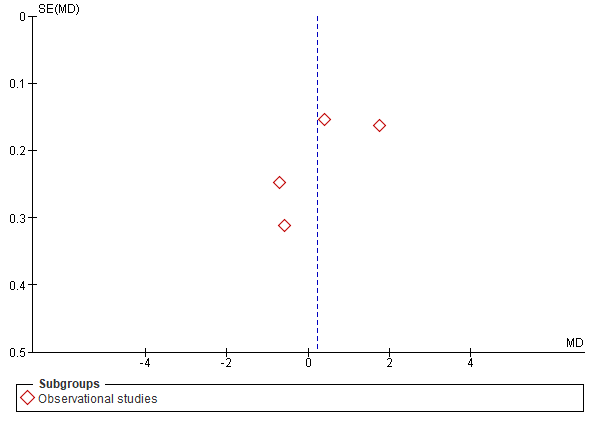** |
| **W: Blood transfusions** | **X: Fluoroscopy time** |
| **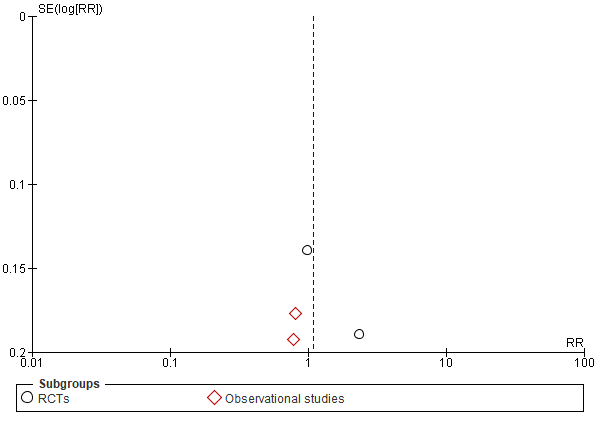** | **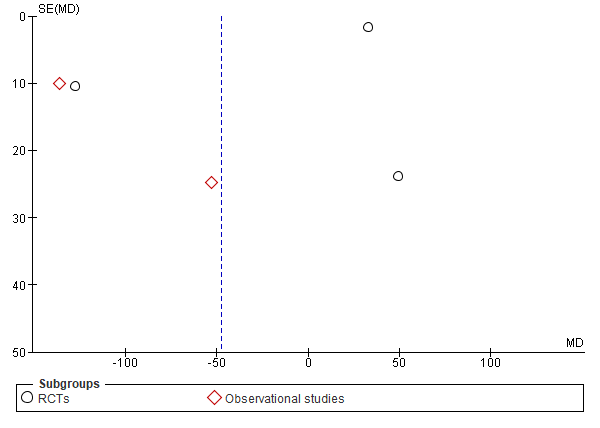** |
| **Y: Tip apex distance** | **Z: Tip apex distance >25 mm** |
| **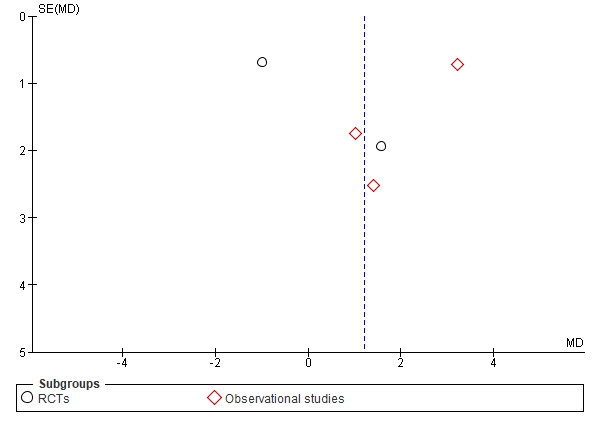** | **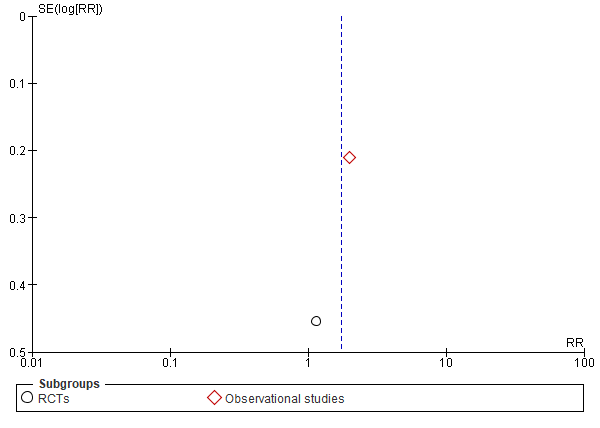** |
| **AA: Femoral neck shortening** | **BB: Neck shaft angle** |
| **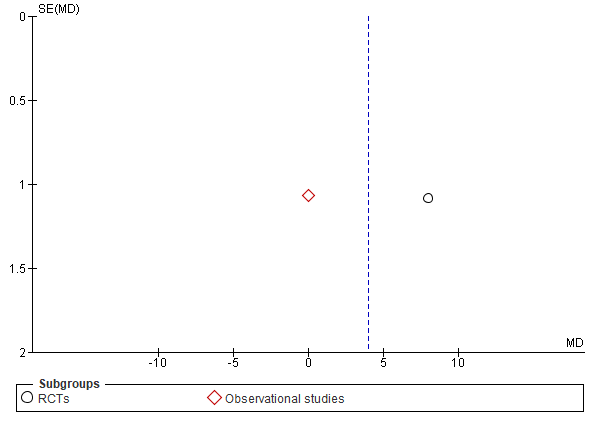** | **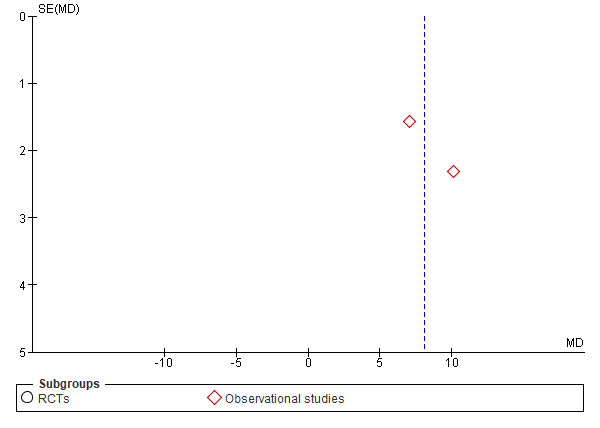** |

**Extramedullary versus intramedullary fixation of unstable trochanteric femoral fractures (AO type 31-A2): a systematic review and meta-analysis**

Archives of Orthopaedic and Trauma Surgery

Miliaan L. Zeelenberg^1#^, MD; A. Cornelis Plaisier^1#^, BSc; Leendert H.T. Nugteren^1^, BSc; Sverre A.I. Loggers^1,2^, MD; Pieter Joosse^2^, MD PhD; Michiel H.J. Verhofstad^1^, MD PhD; Dennis Den Hartog^1^, MD PhD; Esther M.M. Van Lieshout^1^, PhD MSc; STABLE-HIP Study Group*

^1^ Trauma Research Unit Department of Surgery, Erasmus MC, University Medical Center Rotterdam, Rotterdam, The Netherlands

^2^ Department of Surgery, Noordwest Ziekenhuisgroep, Alkmaar, The Netherlands

^#^ Both first authors contributed equally

*Taco Gosens, MD PhD; Johannes H. Hegeman, MD PhD; Suzanne Polinder; Rudolf W. Poolman, MD PhD; Hanna C. Willems; Rutger G. Zuurmond

**Corresponding authors**

Dr. E.M.M. Van Lieshout

Trauma Research Unit Department of Surgery

Erasmus MC, University Medical Center Rotterdam

P.O. Box 2040

3000 CA Rotterdam

The Netherlands

Phone: +31.10.7031050

Mail: [e.vanlieshout@erasmusmc.nl](mailto:e.vanlieshout@erasmusmc.nl)
