# Supplementary material for: Extramedullary versus intramedullary fixation of unstable trochanteric femoral fractures (AO type 31-A2): a systematic review and meta-analysis
Source: Arch Orthop Trauma Surg. 2024 Jan 4;144(3):1189–209. doi: 10.1007/s00402-023-05138-9 (PMC10896832; doi:10.1007/s00402-023-05138-9)
Supplement: Supplementary file 4 — Supplementary file4 (DOCX 351 KB) [file 402_2023_5138_MOESM4_ESM.docx]

**Online resource 4: Additional tables and figures.**

**Table S2: Outcomes measured reported in the included studies**

| **Outcome** | **Corresponding figure** | **Aktselis *et al.* (2014)** [32] | **Andalib *et al.* (2020**) [33] | **Andruszkow *et al.* (2012)** [43] | **Barton *et al.* (2010)** [26] | **Butt *et al.* (2017)** [44] | **Crespo *et al.* (2012)** [45] | **Duymus *et al.* (2019)** [46] | **Garg *et al.* (2022)** [42] | **Grønhaug *et al.* (2022)** [20] | **Knobe *et al.* (2009)** [27] | **Knobe *et al.* (2012)** [47] | **Müller *et al.* (2020)** [48] | **Ovesen *et al.* (2006)** [38] | **Page *et al.* (2016)** [49] | **Pajarinen *et al.* (2005)** [39] | **Parker *et al.* (2017)** [40] | **Pyrhönen *et al.* (2022)** [19] | **Reindl *et al.* (2015)** [28] | **Saleem *et al.* (2020)** [34] | **Sevinç *et al.* (2020)** [29] | **Singh *et al.* (2017)** [35] | **Suh *et al.* (2015)** [50] | **Tao *et al.* (2013)** [41] | **Tucker *et al.* (2018)** [51] | **Verettas *et al.* (2010)** [30] | **Xu *et al.* (2010)** [36] | **Zehir *et al.* (2015)** [37] |
| --- | --- | --- | --- | --- | --- | --- | --- | --- | --- | --- | --- | --- | --- | --- | --- | --- | --- | --- | --- | --- | --- | --- | --- | --- | --- | --- | --- | --- |
| Harris Hip score | 2 | - | - | - | - | - | - | + | + | - | + | + | - | - | - | - | - | - | - | - | + | + | + | + | - | - | - | - |
| Parker mobility score | 3 | + | - | - | - | - | - | - | + | - | - | - | - | - | - | - | - | - | - | - | - | - | - | - | - | - | + | - |
| Lower extremity measure | S2 | - | + | - | - | - | - | - | - | - | - | - | - | - | - | - | - | - | + | - | - | - | - | - | - | - | - | - |
| Pain scores | N.A. | + | - | - | - | - | - | - | - | - | + | - | - | - | - | - | - | - | - | - | - | - | + | - | - | + | - | - |
| Other functional scores and QoL | N.A. | + | - | - | - | - | - | - | - | - | + | + | - | - | - | - | - | - | + | - | - | - | + | - | - | - | - | - |
| Recovery to pre-operative walking ability | S3 | - | - | - | - | - | - | - | - | - | - | - | - | - | - | - | - | - | - | - | - | - | - | - | - | - | + | + |
| Time to full weight bearing | S4 | - | - | - | - | - | - | + | + | - | - | - | - | - | - | - | - | - | - | - | - | - | - | - | - | - | - | - |
| Reoperation | 4 | - | + | - | + | - | - | + | - | + | + | + | + | + | + | - | + | + | - | - | - | - | - | - | + | - | - | + |
| Deep infection | 5 | - | + | - | - | - | - | + | + | - | + | - | - | + | + | + | - | - | + | - | - | - | + | - | - | - | - | + |
| Superficial infection | 6 | - | + | - | - | + | - | + | + | - | - | - | + | - | - | + | - | - | + | + | - | - | + | - | - | + | + | + |
| Nonunion | 7 | - | + | - | - | - | - | + | + | - | - | - | - | - | - | - | + | - | - | + | - | - | - | - | - | - | - | - |
| Cut-out | 8 | + | - | + | - | + | + | + | - | - | + | + | + | + | - | + | + | - | + | - | - | - | - | - | - | - | + | + |
| Peri-implant fracture | 9 | - | - | - | - | + | - | - | + | - | - | + | + | - | - | - | + | - | - | - | - | - | - | - | - | - | - | + |
| Conversion to prosthesis | 10 | - | + | - | - | + | - | + | - | + | + | + | - | + | + | - | + | + | - | - | - | - | - | - | - | - | - | + |
| Implant/fixation failure | 11 | + | + | - | - | - | - | + | - | - | + | - | + | - | - | - | - | - | - | - | - | - | + | - | - | - | + | - |
| Heterotopic ossification | N.A. | - | - | - | - | - | - | - | + | - | - | - | - | - | - | - | - | - | + | - | - | - | + | - | - | - | - | - |
| Leg shortening | S5 | - | - | - | - | - | - | - | - | - | - | + | - | - | - | - | - | - | - | + | - | - | + | - | - | - | + | - |
| Screw migration | S6 | - | - | - | - | - | - | + | - | - | - | + | - | - | - | - | - | - | - | - | - | - | - | - | - | - | - | + |
| Femur shaft fracture | S7 | - | - | - | - | - | - | - | - | - | - | - | - | - | - | - | - | - | - | - | - | - | - | - | - | - | + | + |
| Mortality | 12 | - | - | - | + | - | - | - | - | - | + | + | + | - | - | - | - | - | - | - | - | - | - | - | + | - | + | + |
| Mean time to bone healing | 13 | - | - | - | - | - | - | + | + | - | - | - | - | - | - | - | - | - | - | + | - | - | - | + | - | - | - | + |
| Radiologic quality of reduction | S8 | - | + | - | - | - | - | + | - | - | - | + | + | - | - | - | - | - | - | - | - | - | - | + | - | - | - | - |
| Surgery duration | 14 | + | - | - | - | - | - | + | + | - | + | + | + | - | - | - | - | - | - | + | - | - | - | + | - | + | + | + |
| Hospital stay | S9 | - | - | - | + | - | - | + | + | - | + | + | + | - | + | - | - | - | - | - | - | - | - | + | - | + | + | - |
| Blood loss | S10 | - | - | - | - | - | - | - | + | - | - | - | - | - | - | - | - | - | - | + | - | - | - | + | - | + | + | + |
| Blood transfusion (patients) | S11 | - | - | - | + | - | - | - | - | - | + | + | - | - | - | - | - | - | - | - | - | - | - | - | - | - | + | - |
| Blood transfusion (units per patient) | S12 | - | - | - | - | - | - | + | - | - | + | + | + | - | - | - | - | - | - | - | - | - | - | - | - | + | - | - |
| Fluoroscopy time | S13 | - | - | - | - | - | - | - | - | - | + | + | - | - | - | - | - | - | - | - | - | - | - | + | - | - | + | + |
| TAD | S14 | - | + | - | - | - | - | - | - | - | - | + | + | - | - | - | - | - | + | - | + | - | - | - | - | - | - | + |
| TAD>25mm | S15 | - | - | - | + | - | - | - | - | - | - | - | + | - | - | - | - | - | - | - | - | - | - | - | - | - | - | - |
| Femoral neck shortening | S16 | - | - | - | - | - | - | - | - | - | - | + | - | - | - | - | - | - | + | - | - | - | - | - | - | - | - | + |
| NSA | S17 | - | - | - | - | - | - | + | + | - | - | + | - | - | - | - | - | - | - | - | + | - | - | - | - | - | - | - |

+, reported in article; -, not reported in article; TAD, Tip-Apex Distance; mm, millimeter; NSA, Neck-shaft angle; AMS, Abductor Muscle Strength; N.A., Not available; QoL, Quality of life.

**Table S3: Quality assessment of the included RCT’s using the Cochrane RoB 2 tool**

| **Study** | D1 | D2 | D3 | D4 | D5 | **Overall** |
| --- | --- | --- | --- | --- | --- | --- |
| Aktselis *et al.* (2014) [32] | + | x | + | + | + | **-** |
| Andalib *et al.* (2020) [33] | x | x | x | + | + | **x** |
| Barton *et al.* (2010) [26] | + | + | + | + | + | **+** |
| Garg *et al.* (2022) [42] | + | - | + | + | + | **-** |
| Ovesen et al. (2006) [38] | + | + | + | + | + | **+** |
| Pajarinen et al. (2005) [39] | + | x | - | + | + | **x** |
| Parker et al. (2017) [40] | + | + | - | + | + | **-** |
| Reindl *et al.* (2015) [28] | - | + | + | + | - | **-** |
| Saleem *et al.* (2020) [34] | + | + | + | + | + | **+** |
| Singh *et al.* (2017) [35] | + | - | + | + | + | **-** |
| Tao et al. (2013) [41] | + | + | + | + | + | **+** |
| Verettas *et al.* (2010) [30] | x | + | + | + | + | **x** |
| Xu *et al.* (2010) [36] | + | - | + | + | + | **-** |
| Zehir *et al.* (2015) [37] | + | + | + | + | + | **+** |

**+** Low risk of bias; **-** Some concerns of bias; **x** High risk of bias

Domains: D1, Randomization process; D2, Deviations from intended interventions; D3, Missing outcome data; D4, Measurement of the outcome; D5, Selection of the reported result

**Table S4: Quality assessment of the included observational studies using the MINORS criteria**

| **Study** | Aim | Inclusion | Collection | Endpoints | Assessment | Follow-up period | Loss of  Follow-up | Study size calculation | Control group | Contemporary group | Baseline equivalence | Statistics | **Total** |
| --- | --- | --- | --- | --- | --- | --- | --- | --- | --- | --- | --- | --- | --- |
| Andruszkow *et al.* (2012) [43] | 2 | 2 | 0 | 2 | 0 | 1 | 2 | 0 | 1 | 2 | 1 | 2 | **15** |
| Butt et al. (2017) [44] | 1 | 2 | 0 | 1 | 0 | 0 | 2 | 0 | 2 | 2 | 1 | 2 | **13** |
| Crespo *et al.* (2012) [45] | 1 | 2 | 0 | 2 | 0 | 2 | 1 | 0 | 2 | 2 | 1 | 2 | **15** |
| Duymus *et al.* (2019) [46] | 2 | 2 | 0 | 2 | 0 | 2 | 2 | 0 | 2 | 2 | 1 | 2 | **17** |
| Grønhaug *et al.* (2022) [20] | 2 | 2 | 2 | 2 | 0 | 2 | 1 | 0 | 2 | 2 | 2 | 2 | **19** |
| Knobe *et al.* (2009) [27] | 1 | 2 | 0 | 2 | 2 | 2 | 0 | 0 | 2 | 2 | 1 | 2 | **16** |
| Knobe *et al.* (2012) [47] | 2 | 2 | 2 | 2 | 1 | 2 | 2 | 1 | 2 | 2 | 2 | 2 | **22** |
| Müller *et al.* (2020) [48] | 1 | 2 | 1 | 2 | 0 | 2 | 2 | 0 | 2 | 2 | 2 | 2 | **18** |
| Page *et al.* (2016) [49] | 2 | 2 | 0 | 1 | 0 | 0 | 2 | 0 | 2 | 2 | 1 | 2 | **14** |
| Pyrhönen *et al.* (2022) [19] | 2 | 2 | 2 | 2 | 0 | 2 | 2 | 0 | 2 | 2 | 2 | 2 | **20** |
| Sevinç *et al.* (2020) [29] | 2 | 0 | 2 | 2 | 0 | 2 | 2 | 0 | 2 | 0 | 1 | 2 | **15** |
| Suh *et al.* (2015) [50] | 2 | 1 | 2 | 2 | 0 | 2 | 1 | 0 | 2 | 2 | 1 | 2 | **17** |
| Tucker *et al.* (2018) [51] | 2 | 2 | 1 | 2 | 0 | 2 | 2 | 0 | 2 | 2 | 1 | 2 | **18** |

Total scores range from 0 to 24 points, with lower scores indicating a higher risk of bias.

0, not reported; 1, reported but inadequate; 2, reported and adequate

**
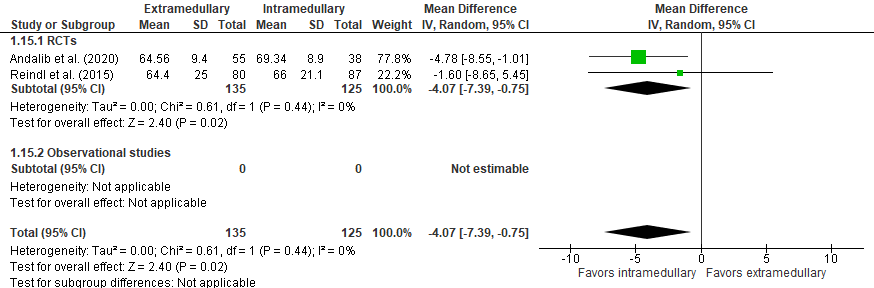
Figure S2:** **Forest plot of Lower Extremity Measure after extramedullary versus intramedullary fixation of AO 31-A2 fractures**

IV, inverse variance; RCT, randomized controlled trial; SD, standard deviation

**
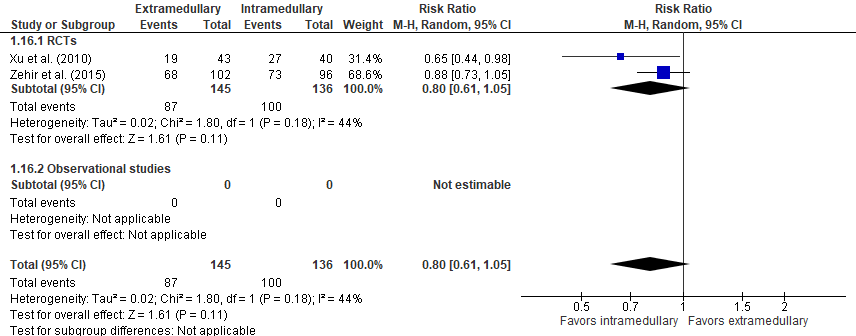
**

**Figure S3: Forest plot of recovery to pre-operative walking ability after extramedullary versus intramedullary fixation of AO 31-A2 fractures**

M-H, Mantel-Haenszel; RCT, randomized controlled trial; SD, standard deviation


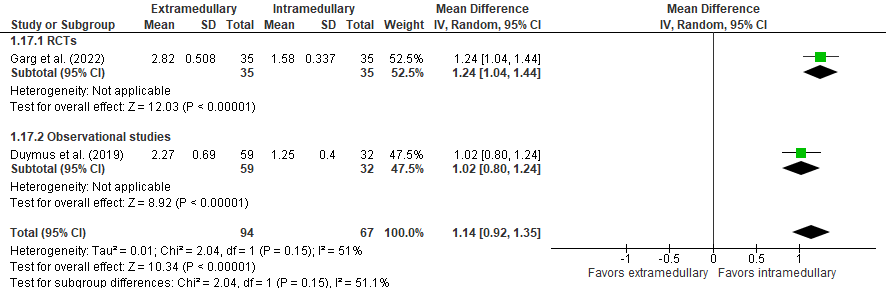


**Figure S4: Forest plot of time to full weight bearing (months) after extramedullary versus intramedullary fixation of AO 31-A2 fractures**


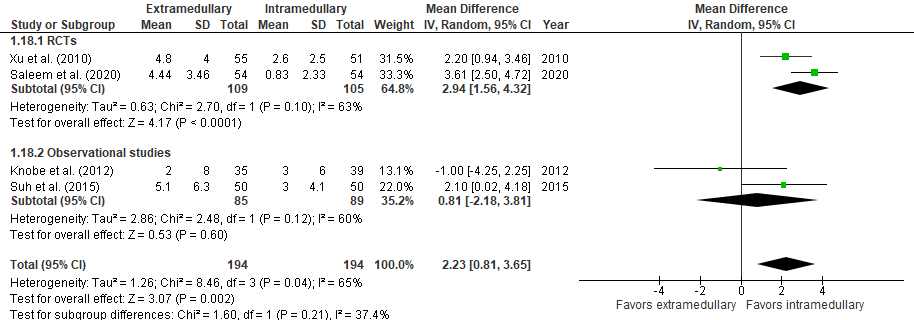


**Figure S5: Forest plot of leg shortening (mm) after extramedullary versus intramedullary fixation of AO 31-A2 fractures**


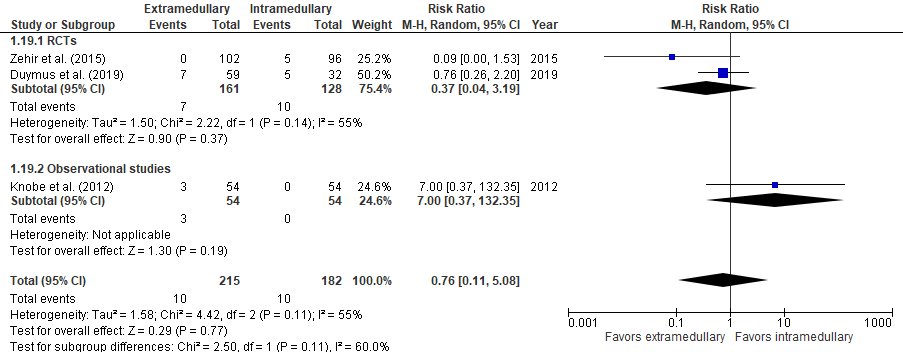


**Figure S6: Forest plot of screw migration after extramedullary versus intramedullary fixation of AO 31-A2 fractures**


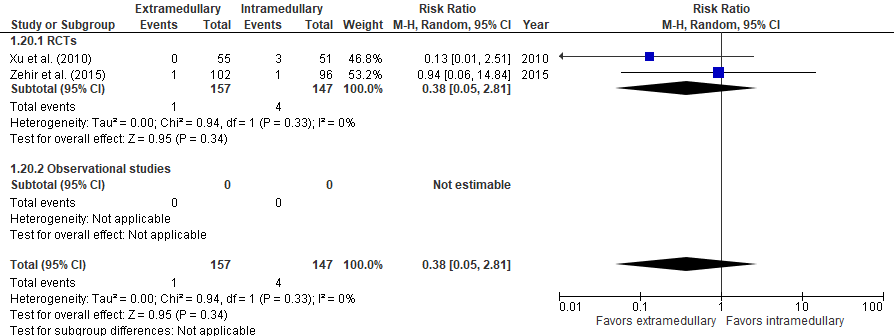


**Figure S7: Forest plot of femoral shaft fractures after extramedullary versus intramedullary fixation of AO 31-A2 fractures**


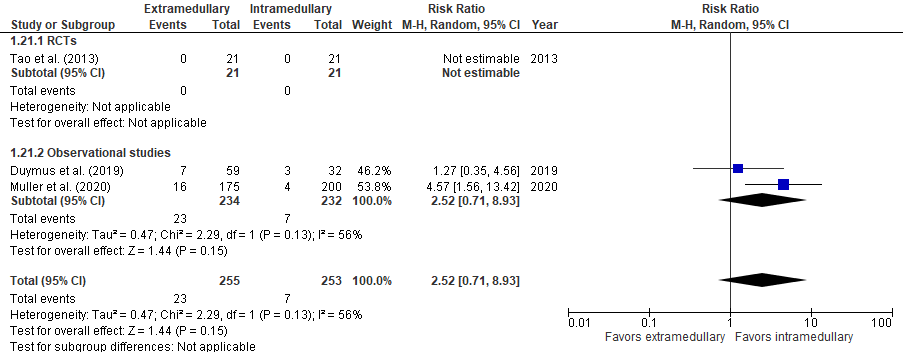


**Figure S8: Forest plot of poor quality of reduction after extramedullary versus intramedullary fixation of AO 31-A2 fractures**


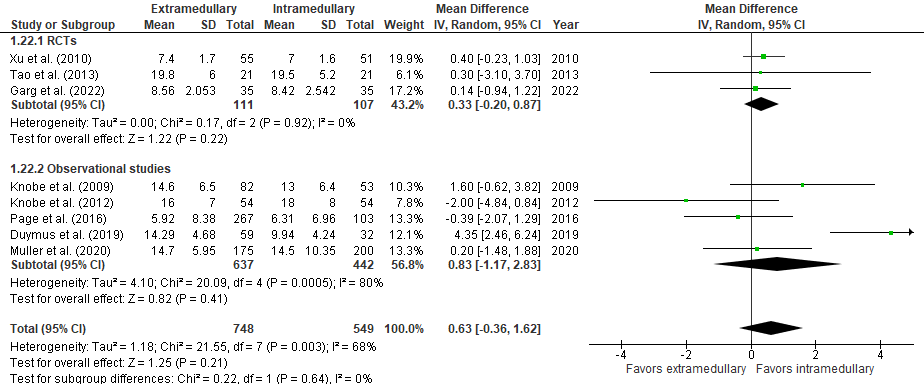


**Figure S9:** **Forest plot of hospital stay (days) after extramedullary versus intramedullary fixation of AO 31-A2 fractures**

Standard deviations for Knobe *et al.* (2009) [27] were imputed.


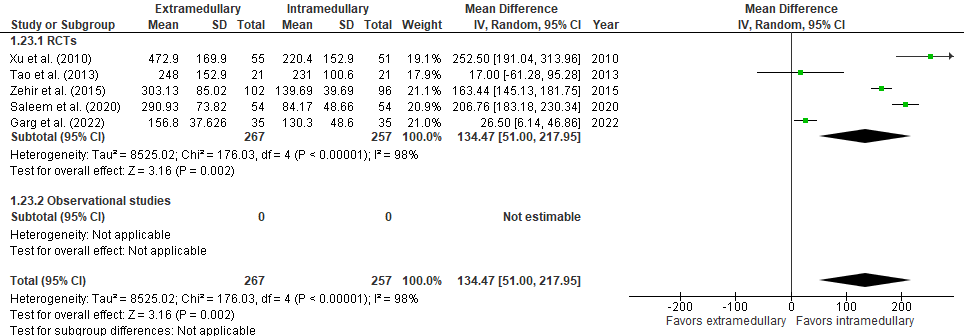


**Figure S10:** **Forest plot of blood loss (mL) in extramedullary versus intramedullary fixation of AO 31-A2 fractures**


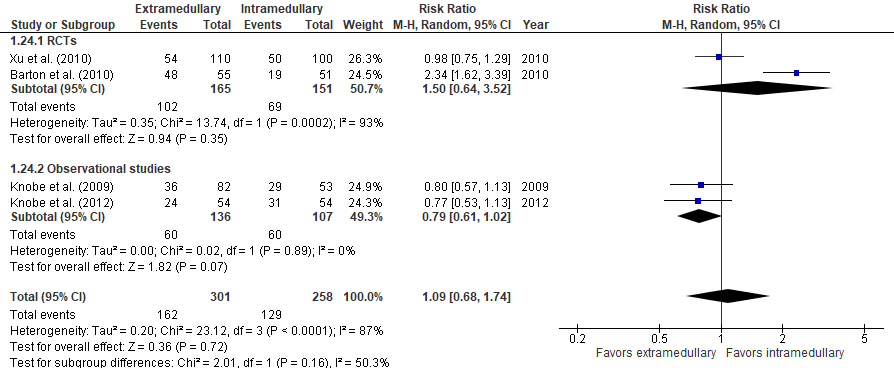
**Figure S11: Forest plot of patients receiving blood transfusion after extramedullary versus intramedullary fixation of AO 31-A2 fractures**

**
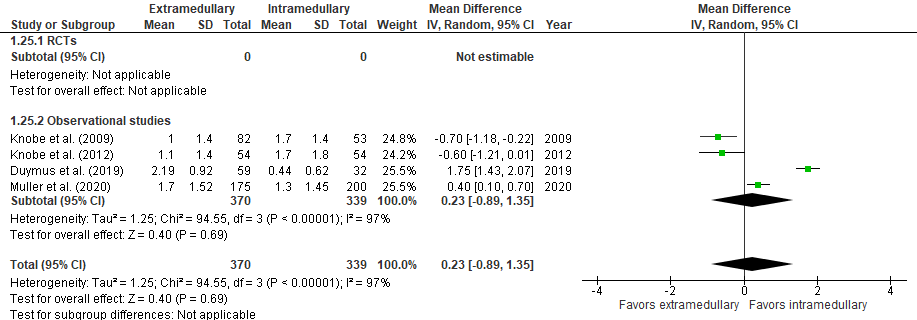
**

**Figure S12: Forest plot of blood replacement units after extramedullary versus intramedullary fixation of AO 31-A2 fractures**

Standard deviations for Knobe *et al.* [27] were imputed.


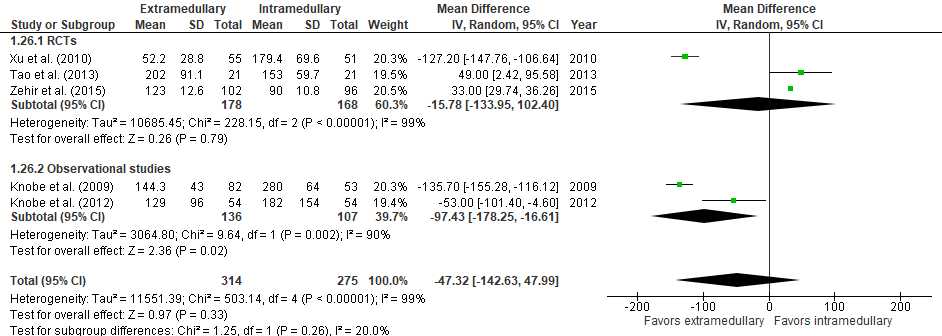


**Figure S13:** **Forest plot of fluoroscopy time (sec) in extramedullary versus intramedullary fixation of AO 31-A2 fractures**

Standard deviations for Knobe *et al.* (2009) [27] were imputed.


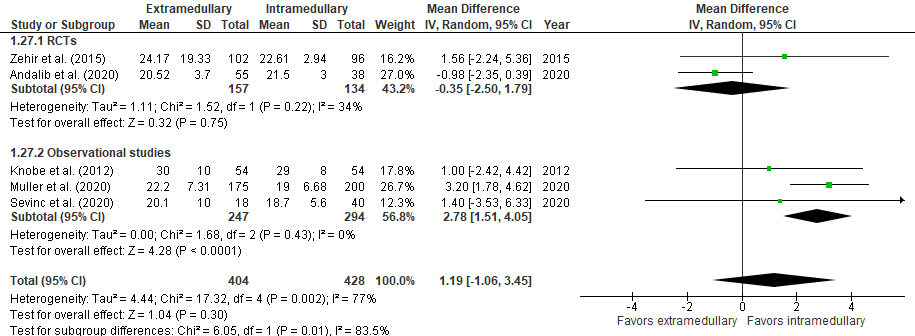


**Figure S14:** **Forest plot of TAD after extramedullary versus intramedullary fixation of AO 31-A2 fractures**

Standard deviations for Sevinc *et al.* (2009) [29] were imputed.


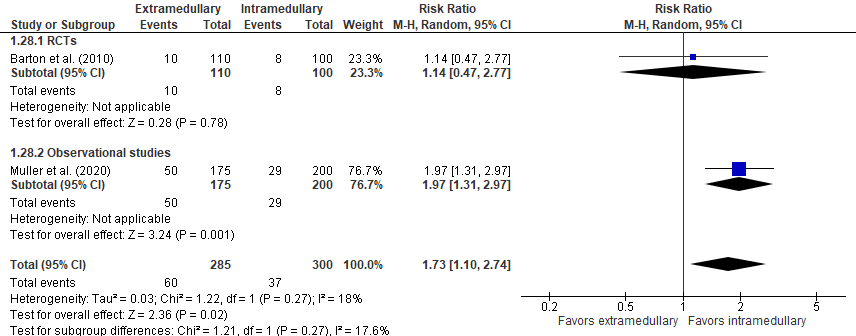


**Figure S15:** **Forest plot of TAD > 25mm after extramedullary versus intramedullary fixation of AO 31-A2 fractures**


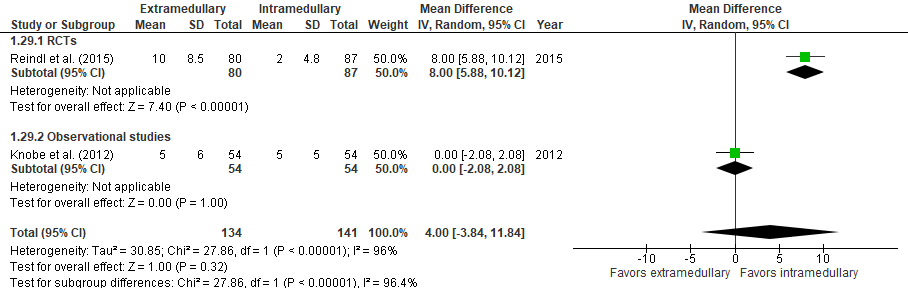


**Figure S16:** **Forest plot of femoral neck shortening (mm) after extramedullary versus intramedullary fixation of AO 31-A2 fractures**


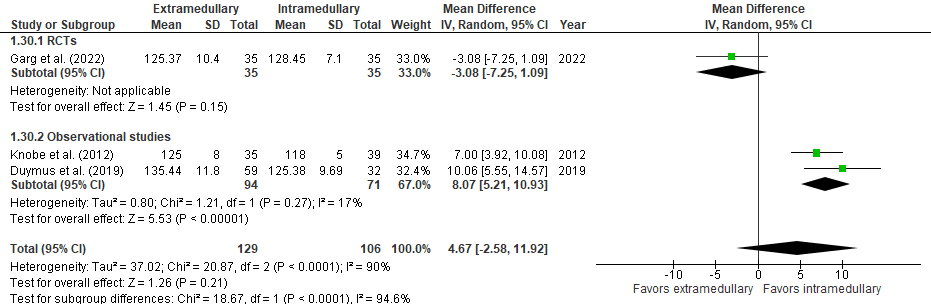


**Figure S17: Forest plot of neck shaft angle (NSA) (degrees) after extramedullary versus intramedullary fixation of AO 31-A2 fractures**

**Extramedullary versus intramedullary fixation of unstable trochanteric femoral fractures (AO type 31-A2): a systematic review and meta-analysis**

Archives of Orthopaedic and Trauma Surgery

Miliaan L. Zeelenberg^1#^, MD; A. Cornelis Plaisier^1#^, BSc; Leendert H.T. Nugteren^1^, BSc; Sverre A.I. Loggers^1,2^, MD; Pieter Joosse^2^, MD PhD; Michiel H.J. Verhofstad^1^, MD PhD; Dennis Den Hartog^1^, MD PhD; Esther M.M. Van Lieshout^1^, PhD MSc; STABLE-HIP Study Group*

^1^ Trauma Research Unit Department of Surgery, Erasmus MC, University Medical Center Rotterdam, Rotterdam, The Netherlands

^2^ Department of Surgery, Noordwest Ziekenhuisgroep, Alkmaar, The Netherlands

^#^ Both first authors contributed equally

*Taco Gosens, MD PhD; Johannes H. Hegeman, MD PhD; Suzanne Polinder; Rudolf W. Poolman, MD PhD; Hanna C. Willems; Rutger G. Zuurmond

**Corresponding authors**

Dr. E.M.M. Van Lieshout

Trauma Research Unit Department of Surgery

Erasmus MC, University Medical Center Rotterdam

P.O. Box 2040

3000 CA Rotterdam

The Netherlands

Phone: +31.10.7031050

Mail: [e.vanlieshout@erasmusmc.nl](mailto:e.vanlieshout@erasmusmc.nl)
